# Immature cell populations and an erythropoiesis gene-expression signature in systemic juvenile idiopathic arthritis: implications for pathogenesis

**DOI:** 10.1186/ar3061

**Published:** 2010-06-24

**Authors:** Claas H Hinze, Ndate Fall, Sherry Thornton, Jun Q Mo, Bruce J Aronow, Gerlinde Layh-Schmitt, Thomas A Griffin, Susan D Thompson, Robert A Colbert, David N Glass, Michael G Barnes, Alexei A Grom

**Affiliations:** 1 Division of Rheumatology, Cincinnati Children's Hospital Medical Center, 3333 Burnet Avenue, Cincinnati, OH 45229, USA; 2 Department of Pathology, Cincinnati Children's Hospital Medical Center, 3333 Burnet Avenue, Cincinnati, OH 45229, USA; 3 Division of Biomedical Informatics, Cincinnati Children's Hospital Medical Center, 3333 Burnet Avenue, Cincinnati, OH 45229, USA; 4 Division of Rheumatology, Children's National Medical Center, 111 Michigan Avenue NW, Washington, DC 20010, USA; 5 National Institute of Arthritis and Musculoskeletal and Skin Diseases, NIH, 10 Center Drive, Bethesda, MD 20892, USA

## Abstract

**Introduction:**

Previous observations suggest that active systemic juvenile idiopathic arthritis (sJIA) is associated with a prominent erythropoiesis gene-expression signature. The aim of this study was to determine the association of this signature with peripheral blood mononuclear cell (PBMC) subpopulations and its specificity for sJIA as compared with related conditions.

**Methods:**

The 199 patients with JIA (23 sJIA and 176 non-sJIA) and 38 controls were studied. PBMCs were isolated and analyzed for multiple surface antigens with flow cytometry and for gene-expression profiles. The proportions of different PBMC subpopulations were compared among sJIA, non-sJIA patients, and controls and subsequently correlated with the strength of the erythropoiesis signature. Additional gene-expression data from patients with familial hemophagocytic lymphohistiocytosis (FHLH) and from a published sJIA cohort were analyzed to determine whether the erythropoiesis signature was present.

**Results:**

Patients with sJIA had significantly increased proportions of immature cell populations, including CD34^+ ^cells, correlating highly with the strength of the erythropoiesis signature. The erythropoiesis signature strongly overlapped with the gene-expression pattern in purified immature erythroid precursors. The expansion of immature cells was most prominently seen in patients with sJIA and anemia, even in the absence of reticulocytosis. Patients with non-sJIA and anemia did not exhibit the erythropoiesis signature. The erythropoiesis signature was found to be prominent in patients with FHLH and in a published cohort of patients with active sJIA, but not in patients with inactive sJIA.

**Conclusions:**

An erythropoiesis signature in active sJIA is associated with the expansion of CD34^+ ^cells, also is seen in some patients with FHLH and infection, and may be an indicator of ineffective erythropoiesis and hemophagocytosis due to hypercytokinemia.

## Introduction

Systemic juvenile idiopathic arthritis (sJIA) differs from other subtypes of JIA (non-sJIA) in many aspects. Although most JIA subtypes have in common the presence of chronic arthritis, patients with sJIA often are first seen with quotidian hectic fevers, an evanescent rash, serositis, and hepatosplenomegaly [1]. In contrast to the other JIA subtypes, marked leukocytosis and thrombocytosis, severe anemia, a marked acute-phase reaction, and hyperferritinemia are usually observed. Other findings supporting a different underlying pathogenic mechanism in sJIA are the absence of reproducible HLA associations, autoreactive B and T cells, and autoantibodies. This has led some investigators to postulate that sJIA represents an autoinflammatory rather than an autoimmune condition [2]. Another striking aspect of sJIA is its strong correlation with the potentially fatal macrophage-activation syndrome (MAS), also known as reactive hemophagocytic lymphohistiocytosis (HLH) [3,4]. Whereas MAS was initially considered a rare complication of sJIA [5], it now has become apparent that as many as 30% to 50% of patients with new-onset sJIA may have subclinical hemophagocytosis [6-8]. MAS shares many features with familial HLH (FHLH), including clinical and laboratory features, but also immunologic abnormalities such as natural killer (NK) cell dysfunction and elevated levels of soluble IL-2Rα and soluble CD163 [8,9]. Both conditions are characterized by a marked increase in serum ferritin levels [10,11]. In our experience, about 50% of patients with new-onset sJIA have elevated serum ferritin levels of > 500 ng/ml [8], which is used as a cut-off in the diagnostic criteria for HLH [12].

Previous studies have shown heterogeneity among patients with new-onset sJIA with regard to peripheral blood mononuclear cell (PBMC) gene expression [13,14]. At least two different subgroups were apparent, with their gene-expression pattern corresponding to serum ferritin levels [14]. These two groups are best distinguished by the presence or absence of an apparent erythropoiesis gene-expression signature that contains multiple erythropoiesis-related genes, including those encoding embryonic hemoglobins that are usually developmentally silenced; in addition, an "innate immune response cluster" was overexpressed in sJIA patients [15]. Although the overexpression of innate immune-response genes was expected, the presence of an erythropoiesis signature in sJIA was surprising because of the reticulocytopenia and absence of apparent erythroid expansion in this condition [16]. Flow cytometry demonstrated that patients with sJIA, on average, have a higher proportion of CD34^+ ^and CD15^+^CD16^- ^immature PBMC subpopulations [14], suggesting that a link exists between these immature cell populations and the characteristic gene-expression signature.

In this article, we demonstrate that a link exists between the expansion of immature PBMC subpopulations and characteristic gene-expression signatures in sJIA and that the erythropoiesis signature also is seen in patients with FHLH and in some patients with systemic lupus erythematosus (SLE) and bacterial infections.

## Materials and methods

### Patients

After written informed consent was provided by their legal guardians, patients were enrolled in an institutional review board-approved prospective multicenter study of gene-expression profiling in childhood arthritis. Patients with physician-diagnosed JIA, based on the 1997 International League of Associations for Rheumatology (ILAR) criteria, were studied before treatment with disease-modifying antirheumatic drugs (DMARDs). The 199 patients (23 with sJIA and 176 without sJIA) were included in this analysis, and 59 healthy controls were also included in the study. Routine laboratory tests, such as white blood cell count (WBC), hemoglobin level (Hgb), platelet count (plt), erythrocyte sedimentation rate (ESR), and C-reactive protein (CRP), were available for the majority of the 199 patients (WBC, 188 of 199; Hgb, 188 of 199; plt, 86 of 199; ESR, 167 of 199; and CRP, 103 of 199) and were obtained either at the time of sampling or within 4 weeks before sampling; these data were not available for control samples. The patients' characteristics are shown in Table 1. Overall, flow-cytometry data were available for 38 of 38 controls (100%) and 149 of 199 JIA patients (75.0%); gene-expression data were available for 29 of 38 controls (64%) and 143 of 199 JIA patients (72.0%). Both flow-cytometry and gene-expression data were available for 29 of 38 controls (76%) and 107 of 199 JIA patients (54%). For an additional part of the study, gene-expression data from PBMC samples of 11 patients with active familial hemophagocytic lymphohistiocytosis (FHLH) were studied; no further clinical data were available for this cohort.

**Table 1:** Baseline patient characteristics of all patients included in the study according to JIA subtype

	Control	Enthesitis related	Extended oligo	Persistent oligo	**RF**^**- **^**poly**	**RF**^**+ **^**poly**	Psoriatic	All non-sJIA	sJIA
Total (*n*)	38	33	8	53	58	15	9	176	23

Flow (*n*)	38	30	5	41	41	15	8	149	14

Expression (*n*)	29	29	7	38	47	14	8	143	21

Flow and expression (*n*)	29	26	4	26	30	14	7	107	12

Age at onset (yr)	10.8^a ^± 5.3	12.4 ± 2.5	4.7 ± 4.7	5.5 ± 4.0	8.1 ± 5.0	10.8 ± 3.0	7.5 ± 4.2	8.2 ± 4.8	5.6 ± 5.0

Onset to baseline (mo)	N/A	7.1 ± 7.8	5.7 ± 3.3	4.4 ± 3.3	8.1 ± 8.3	5.8 ± 9.3	6.7 ± 7.5	6.4 ± 7.0	3.6 ± 5.9

Female (%)	57.8	15.2	100	67.9	75.9	93.3	66.7	64.2	34.8

WBC in 10^9^/L	N/A	6.7 ± 2.0	8.2 ± 2.3	9.0 ± 3.0	8.5 ± 3.6	8.0 ± 2.3	7.6 ± 1.0	8.2 ± 3.0	18.1 ± 9.6^b^

Hgb in g/dl	N/A	13.1 ± 0.9	11.8 ± 0.9	12.2 ± 1.1	12.3 ± 1.1	12.4 ± 1.3	12.5 ± 0.7	12.4 ± 1.1	9.9 ± 1.4^b^

Plt in 10^9^/L	N/A	324 ± 76	368 ± 109	369 ± 96	384 ± 152	375 ± 113	370 ± 96	366 ± 117	548 ± 227^b^

ESR in mm/h	N/A	15 ± 16	23 ± 17	19 ± 15	23 ± 18	31 ± 24	19 ± 17	21 ± 17	89 ± 40^b^

CRP in mg/dl	N/A	1.6 ± 2.2	0.9 ± 1.3	1.2 ± 1.4	1.8 ± 2.8	2.0 ± 2.0	1.0 ± 0.9	1.5 ± 2.1	16.9 ± 14.0^b^

^a^Age at sampling. ^b^*P *< 0.05 (one-way ANOVA and all multiple pairwise comparisons (Tukey)). Numbers represent mean ± standard deviation, unless otherwise indicated. WBC, white blood cell count; Hgb, hemoglobin; ESR, erythrocyte sedimentation rate; CRP, C-reactive protein.

### Sample collection

Sample collection was performed as described previously [13,14] at the time of the baseline visit (before the initiation of DMARD therapy). All further analyses (RNA microarray analysis, flow cytometry, and cytokine measurement) were performed on these samples. In short, peripheral blood was collected by using acid citrate dextrose (ACD) as the anticoagulant. PBMCs were isolated by Ficoll gradient centrifugation, and RNA was immediately stabilized in TRIzol Reagent (Invitrogen, Carlsbad, CA). Aliquots of PBMCs were frozen separately for flow cytometry. Samples were frozen and stored at 80°C at the collecting site before shipment to CCHMC on dry ice.

### RNA processing and microarray analysis

RNA was extracted at CCHMC, purified on RNeasy columns, resuspended in water, and stored at -80°C. Further RNA processing and quality control was performed as described previously [13]. In short, RNA quality was assessed by using the Agilent 2100 Bioanalyzer (Agilent Technologies; Palo Alto, CA). RNA, 100 ng, was labeled by using NuGEN Ovation version 1. RNA samples were randomized into groups of 11, and a universal. standard (pooled PBMC RNA from 35 healthy adult volunteers) was included in each group to measure batch-to-batch variation. Labeled cDNA was hybridized to Affymetrix HG U133 Plus 2.0 GeneChips and scanned with an Agilent G2500A GeneArray scanner. Data were assessed for quality and then imported into GeneSpring GX7.3.1 and pre-processed by using robust multiarray averaging (RMA) followed by normalization of each probe to the median of all samples.

Finally, distance-weighted discrimination was used to address batch-to-batch variations [17]. These GeneChip data are available through NCBI's Gene Expression Omnibus (GEO) [18], series accession GSE21521.

### Flow cytometry

After thawing and washing PBMCs in FACS buffer (PBS with 0.2% BSA), volumes were adjusted to give a concentration of 10^7 ^cells/ml. Cells were then distributed into eight tubes (tubes 1 to 6: 5 × 10^5 ^cells each; tubes 7 and 8, 1 × 10^6 ^cells each). Single-cell suspensions were stained with monoclonal antibodies from BD Biosciences (San Jose, CA): anti-BDCA4 (blood dendritic cell antigen 4)-PE, anti-CD3-Per-CP, anti-CD4-APC, anti-CD8-FITC, anti-CD11c-APC, anti-CD15-PE, anti-CD16-FITC, anti-CD19-APC, anti-CD25-PE, anti-CD33-APC, anti-CD34-FITC, anti-CD45-Per-CP, anti-CD56-APC, anti-CD105-PE, anti-HLA-DR-PE, anti-lineage-cocktail-1 (Lin)-FITC, anti-TCRα/β-FITC, and anti-TCRγ/δ-PE and IgG1 isotype controls. Cells were analyzed in eight separate tubes: (a) unstained cells, (b) isotype controls, (c) anti-CD16-FITC, anti CD15-PE, anti-CD3-PerCP, and anti-CD56-APC; (d) anti-CD8-FITC, anti-CD25-PE, anti-CD3-PerCP, and anti-CD4-APC; (e) anti-TCRα/β-FITC, anti-TCRγ/δ-PE, anti-CD3-PerCP, and anti-CD19-APC; (f) anti-CD34-FITC, anti-CD105-PE, anti-CD45-PerCP, and anti-CD33-APC; (g) anti-Lin-FITC, anti-HLA-DR-PE, and anti-CD11c-APC; and (h) anti-Lin-FITC, anti-BDCA4-PE, and anti-CD11c-APC. Cells were analyzed on a FACSCalibur flow cytometer (BD) by using CELLQuest software. Cells were restricted to a live-cell gate by forward and side-scatter parameters, and cell populations of interest were captured by standardized polygonal gates. The following PBMC subpopulations were analyzed: NK cells (CD3^-^CD56^+^, CD3^-^CD56^+ ^bright, CD3^-^CD56^+ ^dim), T cells (CD3^+^, CD3^+^CD4^+^, CD4^+^CD25^-^, CD4^+^CD25^+^, CD3^+^CD8^+^, CD8^+^CD25^-^, CD8^+^CD25^+^, CD3^+^CD56^+^, CD3^+^TCRα/β, CD3^+^TCRγ/δ), B cells (CD3^-^CD19^+^), myeloid cells (CD15^+^CD16^- ^immature granulocyte, CD15^+^CD16^+ ^mature granulocyte, CD45^+^CD33^+ ^monocyte), dendritic cells (Lin-HLA-DR^+^, Lin-HLA-DR^+^CD11c^+ ^myeloid dendritic cells, Lin-BDCA4^+ ^plasmacytoid dendritic cells), precursor cells (CD34^+^, CD34^+^CD33non-myelomonocytic precursor cells, CD34^+^CD33^+ ^myelomonocytic precursor cells), other cells (CD45^-^CD105^+ ^endothelial cells), and ratios (NK bright-to-dim ratio and CD4:CD8 ratio).

### Cytokine measurements

25 μl of serum from 16 normal controls, 24 enthesitis-related, six oligoarticular, eight RF^- ^polyarticular, and eight systemic JIA patients included in this study was used to measure cytokines with a multiplex bead-based assay (LINCOplex Multiplex Human Cytokine Kit; Millipore, Billerica, MA), according to the manufacturer's recommendations.

### Data analysis

After import into GeneSpring GX 7.3.1 and preprocessing as described before, a supervised analysis was performed by using *t *test or ANOVA (with a Benjamini Hochberg false-discovery rate of 5%) followed by Tukey *post hoc *testing to identify genes with differential expression between predefined groups, where appropriate. Hierarchic clustering of samples and gene lists with the genes selected by supervised analysis was performed by using Pearson correlation. With SigmaPlot 11.0 (SYSTAT Software, San Jose, CA) for the comparison of PBMC proportions among the different groups and controls, one-way ANOVA testing was performed, followed by *post hoc *multiple pairwise comparisons (Tukey), always assuming a significance level of 0.05. For longitudinal comparisons, paired *t *tests (if normally distributed) or signed-rank tests (if not normally distributed) were performed. For the correlation analysis between gene-expression signatures and PBMC subpopulations, Pearson's correlation coefficients were determined. As an indicator of the strength of the expression of the erythropoiesis signature in individual samples, the geometric mean () of the linear expression values of the 67 probe sets in the erythropoiesis signature was calculated and termed the erythropoiesis index. Receiver-operating characteristic (ROC) curve analysis was performed by using SigmaPlot 11.0.

### Signature-overlap analysis

A subset of the GeneChip data (721 B-Lymphoblast, BDCA4^+^, CD105^+^, CD14^+^, CD19^+^, CD33^+^, CD34^+^, CD56^+^, CD4^+^, CD8^+^, and CD71^+^) from a larger data set [19] was imported into GeneSpring 7.3 with RMA preprocessing. Because of limited replicates (two for each cell type), probes sets with a ratio of > 3 (geometric mean of specific cell type to geometric mean of all cell types) were considered expressed in a cell type. Different GeneChips were used for the current analysis (HG U133 plus 2.0) and the imported dataset (HG U133A), and it was determined that only 49 of the 67 erythropoiesis probe sets were present on both arrays.

## Results

### PBMC subpopulations in non-sJIA, sJIA, and controls

In a previous study, by comparing PBMC gene expression between sJIA patients and healthy controls, we identified a group of 67 probe sets that we defined as an erythropoiesis signature [14]. To better understand the origin of this erythropoiesis signature, PBMC phenotyping by flow cytometry was performed. For this purpose, 26 PBMC subpopulations or ratios were compared among the different JIA subtypes. When comparing the percentages of the different PBMC cell populations among patients with non-sJIA, sJIA, and controls, 10 subpopulations or ratios had different mean percentages (ANOVA; *P *< 0.05): CD3^-^CD56^**+**^, CD56^**+ **^dim, CD15^**+**^CD16^-^, CD19^**+**^, CD34^**+**^, CD34^**+**^CD33^-^, CD34^**+**^CD33^**+**^, CD8^**+**^CD25^**+**^, CD56^**+ **^bright-to-dim ratio and CD4:CD8 ratio (Table 2). Most of the differences were observed when comparing the sJIA patients with either (a) patients with non-sJIA or (b) controls, as shown by Tukey's pairwise multiple comparison procedure (*post hoc *testing). An expansion of the following immature PBMC subpopulations in the sJIA group was observed when compared with non-sJIA: CD15^**+**^CD16^- ^immature neutrophils, CD34^**+**^, CD34^**+**^CD33^-^, and CD34^**+**^CD33^**+ **^precursor cells. To determine whether the various immature cell populations across all samples correlate with each other, we compared the percentages of immature cell populations with each other (Pearson's correlation coefficient): CD15^**+**^CD16^- ^and CD34^**+ **^(*r *= 0.28), CD15^**+**^CD16^- ^and CD34^**+**^CD33^- ^(*r *= 0.28), CD15^**+**^CD16^- ^and CD34^**+**^CD33^**+ **^(*r *= 0.18), CD34^**+ **^and CD34^**+**^CD33^- ^(*r *= 0.96), CD34^**+ **^and CD34^**+**^CD33^**+ **^(*r *= 0.68), CD34^**+**^CD33^- ^and CD34^**+**^CD33^**+ **^(*r *= 0.45).

**Table 2:** PBMC subpopulations for which significant differences existed between the controls, patients with JIA other than sJIA, and patients with systemic arthritis

PBMC subpopulation	Percentage of PBMC				Pairwise multiple comparison		
	
	1. Controls	2. non-sJIA	3. sJIA	ANOVA	**1. vs. 2**.	**1. vs. 3**.	**2. vs. 3**.
CD3^-^CD56^+^	6.1 ± 3.9	7.4 ± 4.0	4.7 ± 1.7	0.02	NS	NS	< 0.05

CD56^+ ^dim	5.5 ± 3.5	6.7 ± 3.9	3.7 ± 1.4	0.009	NS	NS	< 0.05

CD15^+^CD16^-^	0.05 ± 0.03	0.06 ± 0.05	0.18 ± 0.19	< 0.001	NS	< 0.05	< 0.05

CD8^+^CD25^+^	0.46 ± 0.26	0.78 ± 0.60	0.70 ± 0.40	0.005	< 0.05	NS	NS

CD19^+^	16.8 ± 6.6	13.2 ± 5.8	11.7 ± 6.6	0.002	< 0.05	< 0.05	NS

CD34^+^	0.08 ± 0.04	0.08 ± 0.05	0.16 ± 0.11	< 0.001	NS	< 0.05	< 0.05

CD34^+^CD33^-^	0.05 ± 0.03	0.06 ± 0.04	0.11 ± 0.09	< 0.001	NS	< 0.05	< 0.05

CD34^+^CD33^+^	0.03 ± 0.02	0.02 ± 0.02	0.05 ± 0.03	< 0.001	< 0.05	NS	< 0.05

NK bright/dim ratio	0.14 ± 0.09	0.13 ± 0.12	0.28 ± 0.16	< 0.001	NS	< 0.05	< 0.05

CD4:CD8 ratio	2.20 ± 0.7	2.14 ± 0.7	2.81 ± 0.9	0.006	NS	< 0.05	< 0.05

Differences were analyzed with one-way ANOVA followed by Tukey's pairwise multiple comparison, assuming a significance level of *P *< 0.05. PBMCs, peripheral blood mononuclear cells; JIA, juvenile idiopathic arthritis; sJIA, systemic JIA; non-sJIA, JIA other than sJIA.

### Correlation of an erythropoiesis signature and innate immune response signature with immature precursor cells

The previously mentioned and reported erythropoiesis signature consists of 67 probe sets, many representing fetal and embryonic hemoglobins (hemoglobins δ, μ, γ, and θ), erythrocyte structural proteins (erythrocyte membrane protein band 4.2), surface proteins (glycophorins A, C), transporter proteins (solute carrier family 22, member 4; solute carrier family 25, member 37), enzymes (2,3-bisphosphoglycerate mutase, carbonic anhydrase I, δ-aminolevulinate synthase 2), and transcription factors (Kruppel-like factor 1) (see Additional file 1) [14]. Because this signature could potentially be produced by immature PBMC subpopulations, such as erythrocyte precursors, we wanted to determine whether this signature is associated with an expansion of immature PBMC subpopulations. We defined the erythropoiesis index as the geometric mean of the normalized expression values of the 67 probe sets contained in the erythropoiesis signature, signifying the strength of the erythropoiesis signature in individual samples or patients. The erythropoiesis index correlated significantly with the percentage of immature cell populations of PBMCs (Table 3): CD34^+^, CD34^+^CD33^-^, and CD15^+^CD16^-^; no significant correlation appeared for other PBMC subpopulations (data not shown). A similarly large correlation was observed for a previously described "innate immune response cluster" [14] with the following cell populations: CD34^+ ^(*r *= 0.64), CD34^+^CD33^- ^(*r *= 0.60) and CD15^+^CD16^- ^(*r *= 0.47), CD34^+^CD33^+ ^(*r *= 0.46), indicating that these cells are also highly associated with this gene-expression signature.

**Table 3:** Pearson correlation coefficients of the geometric mean of the erythropoiesis gene-expression signature values and different PBMC subpopulations across all available samples (*n *= 152)

PBMC subpopulation/ratio	*r *(Pearson)	Correlation *P *value
CD34^+^	0.48	< 0.001

CD34^+^CD33^-^	0.45	< 0.001

CD15^+^CD16^-^	0.45	< 0.001

CD34^+^CD33^+^	0.36	< 0.001

NK bright/dim ratio	0.38	< 0.001

CD15^+^CD16^+^	0.38	0.04

|r| > 0.5 represents a large degree of correlation, 0.3 < |r| < 0.5 represents a moderate degree of correlation and 0.1 < |r| < 0.3 represents a small degree of correlation; |r| < 0.1 represents a lack of correlation (upper panel). Only PBMC subpopulations are shown for which *P *was < 0.05. A low *P *value indicates that the observed correlation coefficient is due to a statistically significant relation between the two variables.

### The erythropoiesis signature overlaps with the gene-expression patterns from purified CD71^+ ^erythroid precursors

At the initiation of these studies, we did not anticipate finding an erythropoiesis signature; therefore, this study was not designed to measure erythrocyte precursor cells directly. Further to delineate the cellular origin of the observed erythropoiesis signature, we assessed its overlap with the gene-expression signatures that we derived from a publicly available dataset derived from isolated cell populations [19]. The publicly available dataset was generated by using Affymetrix U133A microarrays, whereas our data were generated by using U133 Plus 2.0 GeneChips. Of the 67 probe sets defining the erythropoiesis signature, only 49 were present in the U133A arrays. As shown in Table 4, of the 49 probe sets, 39 (79%) were found in the signature of CD71^+ ^immature erythroid precursor cells. A smaller overlap was observed with CD105^+ ^mesenchymal stem/endothelial precursor cells (36.7%), and hematopoietic CD34^+ ^cells (8%). In contrast, essentially no overlap was found with the signatures of BDCA4^+^, CD14^+^, CD19^+^, CD33^+^, CD56^+^, CD4^+^, or CD8^+ ^cells, suggesting that the most likely origin of the erythropoiesis signature was immature erythroid precursors. These results were also investigated through hierarchic clustering (see Additional file 2).

**Table 4:** Overlap between erythropoiesis signature and cell-specific signatures

Cell type	**Probe sets in cell-specific signature**^**a**^	**Overlap with erythropoiesis signature**^**b**^	**Overlap observed**^**c**^	**Overlap expected**^**d**^
CD4^+ ^T lymphocytes	211	0	0	0.9%

CD8^+ ^T lymphocytes	252	0	0	1.1%

CD14^+ ^monocytes	447	0	0	2.0%

CD19^+ ^B lymphocytes	272	1	2.0%	1.2%

CD56^+ ^NK cells	265	0	0	1.2%

CD33^+ ^myeloid lineage	448	0	0	2.0%

CD34^+ ^stem cells	227	4	8.2%	1.0%

CD105^+ ^mesenchymal stem cells/endothelial precursors	360	18	36.7%	1.6%

CD71^+ ^erythroid precursors	563	39	79.6%	2.5%

BDCA4^+ ^dendritic cells	372	0	0	1.7%

^a^Determined by using the HG U133A GeneChip.

^b^Number relative to the 49 probe sets from the erythropoiesis signature that are present on the U133A GeneChip.

^c^(Number of probe sets overlapping with erythropoiesis signature)/(49(total of probe sets in the erythropoiesis signature)) × 100.

^d^(Number of probe sets in the signature) × (49(total of probe sets in the erythropoiesis signature))/(22,283 total of probe sets on the U133A GeneChip) × 100.

### Expansion of immature CD34^+ ^cells mainly in sJIA with anemia

**Figure 1:**
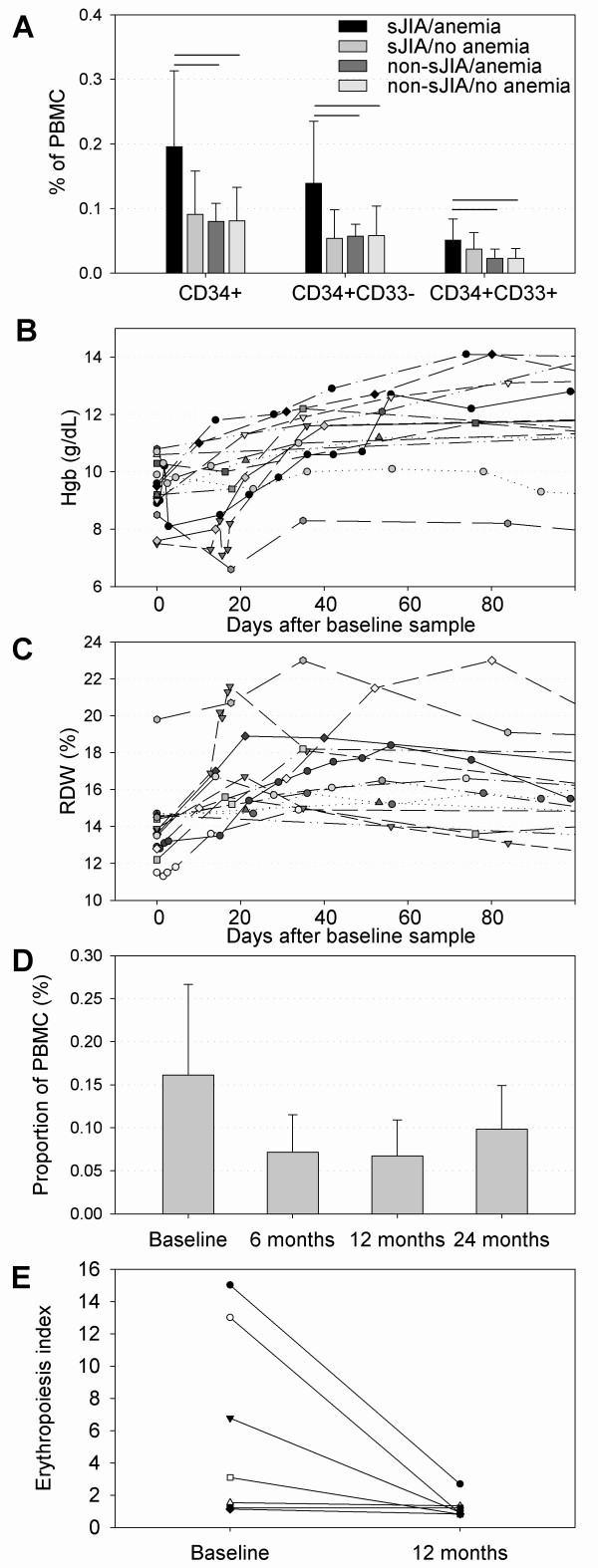
**Comparison of CD34^+ ^precursor cell populations and time course of Hgb, RDW, CD34^+ ^cell proportions and erythropoiesis index**. **(a) **Concentration of circulating CD34^+ ^precursor cells expressed as proportion of PBMCs in patients with sJIA and other JIA subtypes with or without anemia. *P < 0.05 (Student's *t *test). **(b) **Time course of individual hemoglobin concentration after the baseline sample and initiation of treatment in 12 patients with sJIA. A significant increase was noted between baseline and time points 30, 60, and 90 days (paired *t *test, *P *< 0.05). **(c) **Time course of individual red-cell distribution width (RDW) after the baseline sample and initiation of treatment in 12 patients with sJIA. A significant increase was seen between baseline and time points 30 and 60 days (paired *t *test, *P *< 0.05). **(d) **Decrease in CD34^+ ^precursor cell proportions after initiation of treatment in 13 patients with sJIA. A significant decrease was found between baseline and time points 6 months and 12 months (*t *test, *P *< 0.05). **(e) **Decrease in the erythropoiesis index (geometric mean of the erythropoiesis signature expression) between baseline and 12 months later in seven patients for whom longitudinal gene-expression data were available (Signed-rank test, *P *< 0.05). sJIA, systemic juvenile idiopathic arthritis; non-sJIA, JIA other than sJIA; PBMCs, peripheral blood mononuclear cells; Hgb, hemoglobin; RDW, red cell distribution width.

One of the features of sJIA is severe anemia with reticulocytopenia [16]; therefore, the presence of an erythropoiesis signature was surprising. Because the erythropoiesis signature correlated with the PBMC percentage of immature cell subpopulations (CD34^+^, CD34^+^CD33^-^, CD34^+^CD33^+^), a further analysis was performed to determine whether the expansion of these cell populations is related to the presence or absence of anemia (hemoglobin < 11 g/dl). For 21 patients with sJIA and for 166 patients with non-sJIA, hemoglobin levels were available. The groups had the following characteristics (*n*; Hgb mean ± standard deviation; ESR, mean ± standard deviation): (1) sJIA with anemia (*n *= 18; Hgb, 9.4 ± 1.0 g/dl; ESR, 96 ± 36 mm/h); (2) sJIA without anemia (*n *= 3; Hgb, 11.9 ± 1.0 g/dl; ESR, 62 ± 49 mm/h); (3) non-sJIA with anemia (*n *= 21; Hgb, 10.5 ± 0.4 g/dl; ESR, 33 ± 18 mm/h); and (4) non-sJIA without anemia (*n *= 145; Hgb, 12.7 ± 0.9 g/dl; ESR, 18 ± 18 mm/h). Patients with sJIA and anemia had a significantly larger proportion of circulating CD34^+^, CD34^+^CD33^-^, and CD34^+^CD33^+ ^cells than did individuals with non-sJIA with or without anemia (Figure 1a). Although the values did not reach statistical significance, patients with sJIA and anemia trended toward higher percentages of CD34^+ ^cell subpopulations than did patients with sJIA without anemia, suggesting that the expansion of these immature PBMC subpopulations was rather specific to individuals with sJIA and anemia. This statistical analysis was restricted because of the low number of patients with sJIA who did not have anemia (*n *= 3).

### Evidence for the absence of reticulocytes in the peripheral blood of patients with systemic arthritis

As mentioned earlier, this study was not designed to measure directly the erythrocyte precursor cells, including reticulocytes. Instead, we used the red-cell distribution width (RDW) as a surrogate marker for the presence of reticulocytosis, as it is well recognized that the RDW highly correlates with the reticulocyte count [20]. These data were available for 12 patients with sJIA in this study. A significant degree of anemia at the baseline visit rapidly and significantly improved after treatment (paired *t *test; baseline vs. 30 days, *P *= 0.002; baseline vs. 60 days, *P *< 0.001; baseline vs. 90 days, *P *= 0.01; the test results closest to 30, 60, and 90 days, respectively, were used) (Figure 1b). The patients with sJIA had a normal RDW at the baseline visit (before receiving any treatment) but experienced a rapid, and significant, increase in the RDW, suggesting the development of reticulocytosis, after initiation of treatment. This decreased at later times (paired *t *test; baseline vs. 30 days, *P *< 0.001; baseline vs. 60 days, *P *= 0.02; baseline vs. 90 days, *P *= 0.11; the test results closest to 30, 60, and 90 days, respectively, were used) (Figure 1c). Concurrently, the percentage of circulating CD34^+ ^precursor cells also decreased within 6 to 12 months (*t *test; baseline vs. 6 months, *P *= 0.04; baseline vs. 12 months, *P *= 0.02; baseline vs. 24 months, *P *= 0.275) (Figure 1d).

### Erythropoiesis signature in systemic arthritis with anemia but not in other JIA subtypes with anemia

**Figure 2:**
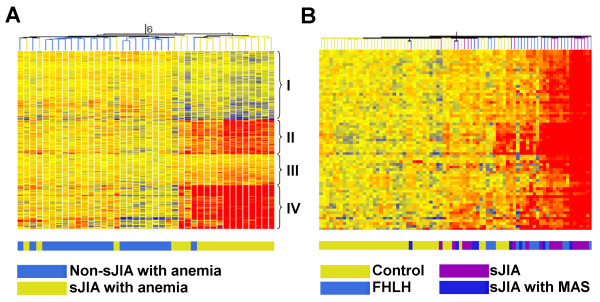
**Differentially expressed probe sets and hierarchic clustering in patients with sJIA and anemia compared with patients with non-sJIA and anemia and hierarchic clustering of controls, patients with sJIA, and those with FHLH**. **(a) **Gene expression of 671 probes that are differentially expressed between 18 patients with systemic arthritis and anemia and 22 patients with other subtypes of JIA and anemia. Genes and samples are clustered by using hierarchic clustering. Four clusters are designated with Roman numerals. **(b) **Gene-expression pattern of 67 probes ("erythropoiesis signature") among healthy controls, patients with familial HLH, systemic arthritis without MAS, and systemic arthritis with MAS. Samples are clustered by using euclidean distances.

Because we identified PBMC subpopulation differences between patients with or without anemia, and to expand our analysis, we looked at gene-expression differences among sJIA and non-sJIA, with and without anemia. Similarly, when comparing gene expression between patients with sJIA with anemia and non-sJIA patients with anemia, 671 genes were differentially expressed (*t *test, 5% FDR [see Additional file 3]). With hierarchic clustering, similar gene clusters emerged, with cluster II and IV being most coherent and upregulated (Figure 2a). A strong segregation of the sample tree was noted, with sJIA patients separating from other JIA patients. Cluster IV (162 genes) corresponded to the previously observed erythropoiesis expression signature, as it contained 52 of the 67 probe sets contained in the initially described erythropoiesis signature [14]. No clustering was observed when comparing patients with sJIA with anemia with patients with sJIA without anemia. The strength of the expression of the erythropoiesis signature in PBMCs was examined in seven patients in whom longitudinal data were available. A marked and significant decrease in the erythropoiesis index between baseline and 12 months later was observed (Figure 1e) (*P *= 0.02, Signed Rank test).

### Overlap with gene expression in PBMC in FHLH

Given the clinical and laboratory similarities between sJIA, MAS, and HLH, we attempted to determine whether a similar erythropoiesis signature was present in patients with FHLH. PBMC-derived RNAs from 11 patients with FHLH were studied for comparison. Hierarchic clustering of the 67 probes contained in the erythropoiesis signature by using samples from patients with FHLH, sJIA, sJIA with MAS, and healthy controls resulted in a partially homogenous cluster with the sJIA and FHLH samples clustering together (Figure 2b), suggesting activation of similar pathways.

### The erythropoiesis signature in other sJIA cohorts

**Figure 3:**
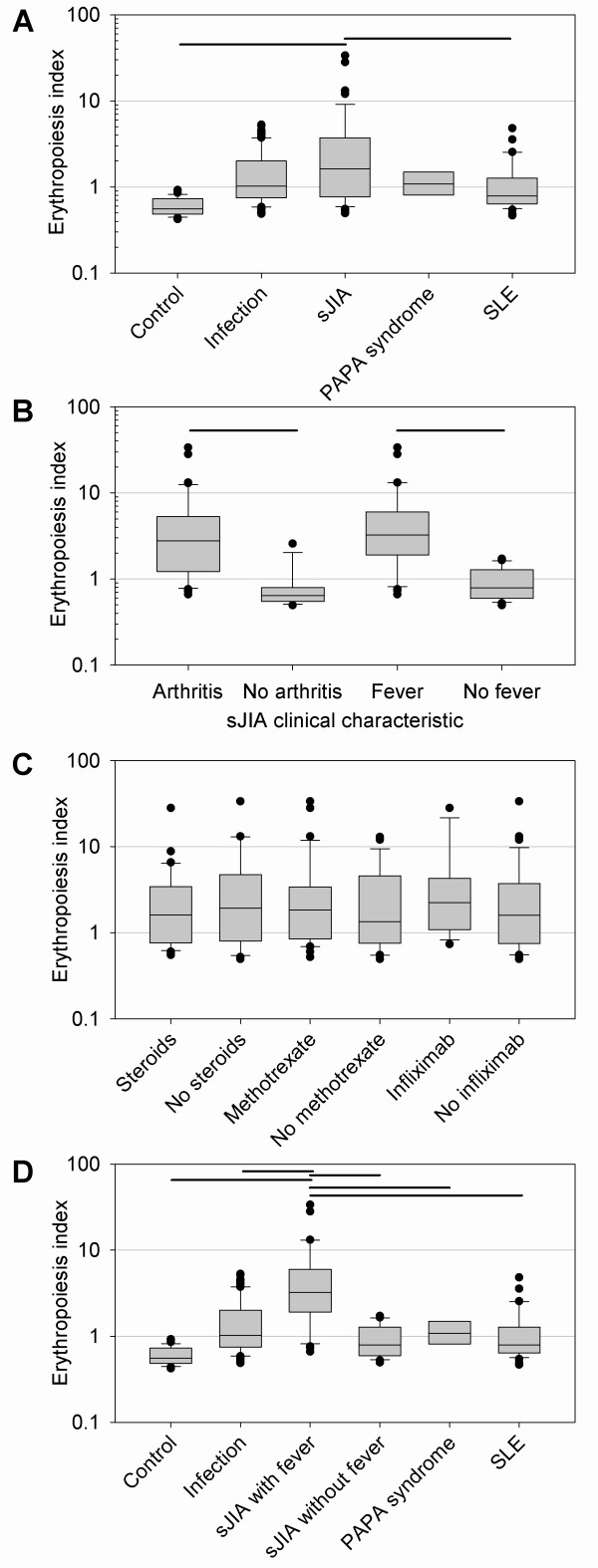
**Erythropoiesis signature in a published cohort of healthy controls, and in patients with sJIA, bacterial infections, PAPA syndrome, and SLE**. Gene-expression data published by Allantaz *et al. *[21] were retrieved from the Gene Expression Omnibus (GEO) database. Forty-nine probe sets were identical between the Affymetrix U133A (used by Allantaz *et al*.) and the U133 Plus 2.0 arrays (used by our group) and part of the erythropoiesis signature. The geometric mean of the linear expression values of these 49 probe sets was calculated for the individual samples, and the mean for the corresponding groups was calculated. **(a) **Comparison of the mean of the modified erythropoiesis indices according to the disease. **(b) **The mean of the modified erythropoiesis indices comparing sJIA with or without fever and with or without arthritis. **(c) **The mean of the modified erythropoiesis indices comparing patients with sJIA receiving or not receiving steroids, methotrexate, or infliximab. **(d) **Comparing the mean of the modified erythropoiesis indices according to disease, subdividing patients with sJIA into those with and those without fever. Horizontal bars indicate *P *< 0.05 (if comparing only two groups with Student's *t *test; if comparing more than two groups, ANOVA and *post hoc *Tukey testing).

To determine how specific the erythropoiesis signature was for sJIA, we expanded our analysis to previously published gene-expression data of a non-overlapping cohort. Studies by Allantaz *et al. *[21] examined PBMC gene expression in patients with active and inactive sJIA and other diseases, including bacterial infections, systemic lupus erythematosus (SLE), and PAPA syndrome by using Affymetrix U133A and U133B GeneChips [21]. We used this dataset (accessed via the GEO database; records GSE8650 and GSE6269) to determine the presence or absence of the erythropoiesis signature. Of the 67 probe sets, 49 contained in the erythropoiesis signature (determined on the Affymetrix U133 Plus 2.0 arrays) were also contained on the U133A GeneChip. We used the modified erythropoiesis index (the geometric mean of the normalized expression values of the 49 probe sets that were available) for further analyses. Comparing the mean of the erythropoiesis indices among the different groups showed that the highest levels of expression of the erythropoiesis signature were observed in patients with sJIA, with statistically significant differences between (a) sJIA and controls, and (b) sJIA and SLE (Figure 3a). In an effort to understand which clinical characteristics were relevant for the erythrocyte signature, we separated patients based on the absence or presence of fever, arthritis, and medical treatment provided in the GEO database. When comparing patients with sJIA with fever with patients with sJIA without fever, and patients with sJIA with arthritis with patients with sJIA without arthritis, a significantly increased expression of the erythropoiesis signature in patients with active sJIA (with fever or arthritis) was observed (Figure 3b). However, no difference in the expression of the erythropoiesis signature was found, whether or not patients were treated with steroids, methotrexate, or infliximab (Figure 3c). When comparing the erythropoiesis-signature expression across all groups and subdividing sJIA into those patients with or without fever, the expression is significantly increased in patients with active sJIA (with fever) when compared with controls, other diseases, and inactive sJIA (without fever) (Figure 3d). To analyze further the discriminating strength of the erythropoiesis index, we performed ROC curve analyses. An area under the curve (AUC) of 1 indicates a perfect diagnostic test, whereas an AUC of 0.5 indicates a test result no better than chance. The AUC of the comparison active sJIA (with fever) versus bacterial infection was 0.82 [see Additional file 4]. The AUC of the comparison active sJIA (with fever) versus "inactive" sJIA (no fever) was 0.92 [see Additional file 4]. The large area under the curve indicates that, in this cohort, the erythropoiesis index was able to discriminate well between patients with active sJIA and bacterial infection and even better between patients with active sJIA and inactive sJIA. Nevertheless, a number of patients with bacterial infections also had an elevated erythropoiesis index, again indicating that the signature was not entirely unique to patients with sJIA.

### Erythropoiesis signature and serum cytokines

**Figure 4:**
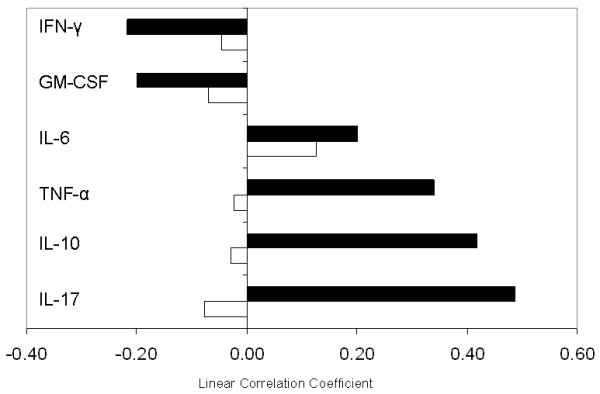
**Pearson correlation coefficients of the "erythropoiesis index" (geometric mean of the linear expression values of the 67 probes in the erythropoiesis signature) and several cytokine protein levels**. The "erythropoiesis index" was correlated with cytokine protein levels across all available samples. A correlation coefficient > 0.5 represents a large degree of correlation; a coefficient between 0.5 and 0.3 represents moderate correlation; a coefficient between 0.3 and 0.1 shows a small degree of correlation; and correlation < 0.1 represents a lack of correlation. (Open bar) Correlation across all samples, 16 normal controls, 24 enthesitis-related, six oligo, eight poly RF-, and eight systemic JIA. (Closed bar) Correlation in systemic JIA.

Because IL-6 has been implicated in the development of anemia in systemic JIA, we assessed the degree of correlation between the erythropoiesis signature and serum levels of various cytokines, including IL-6. Serum samples from 16 normal controls, 24 enthesitis-related, six oligoarticular, eight polyarticular RF^-^, and eight sJIA patients were used in this part of the study. As shown In Figure 4, when all JIA samples were combined, no moderate or strong correlations were observed. When patients with sJIA were analyzed separately, we observed a moderate correlation with IL-17 and IL-10, but only a mild correlation with IL-6. The eight patients with sJIA included in this analysis were representative of the entire sJIA cohort in this study (Hgb mean, 9.5 g/dl; standard deviation, 1.3; CRP mean, 15.3 mg/dl; standard deviation, 8.6; ESR mean, 89 mm/h; standard deviation, 51). Serum IL-6 levels in patients with sJIA ranged between 11.9 and 481.2 pg/ml (mean, 179.4 pg/ml; standard deviation, 180.1 pg/ml).

### Erythroid precursors in the bone marrow of patients with systemic JIA and anemia

**Figure 5:**
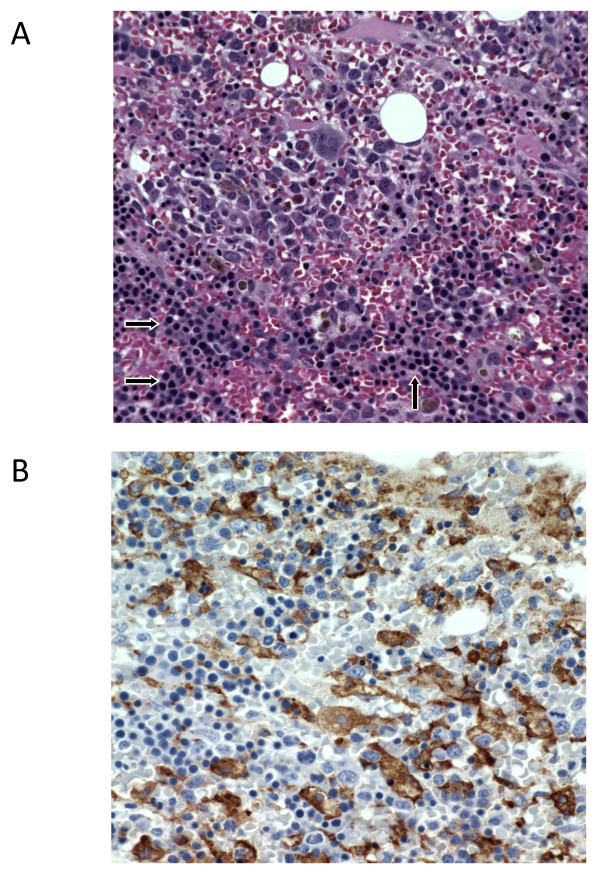
**Bone marrow biopsy from a patient with new-onset systemic JIA**. **(a) **H&E staining showing hypercellular bone marrow with prominent accumulations of nucleated erythroblasts (arrows). **(b) **Immunostaining with monoclonal antibodies specific for CD163. Brown staining identifies CD163^+ ^cells (some are hemophagocytic). CD163^+ ^macrophages are very rare in a normal bone marrow (not shown).

In two of the patients included in this study, bone marrow biopsy was performed as a part of the initial diagnostic evaluation to rule out malignancy (these patients had relatively low Hb levels: 7.2 and 7.8 g/dl). Both patients were found to have hypercellular bone marrow with mild expansion of erythroid lineage (Figure 5). These patients also had moderate expansion of CD163^+ ^histiocytes, a phenomenon viewed by some [7] as early stages of MAS.

## Discussion

The concurrent collection of RNA microarray data and detailed PBMC cell phenotyping provided powerful means to determine associations between different gene-expression signatures and PBMC subpopulations. This is based on the fact that differences in gene-expression patterns seen in the peripheral blood are due not only to the up- and downregulation of gene expression but also to the over- or underrepresentation of certain cell populations. The data presented in this study demonstrate that specific gene-expression signatures in sJIA are associated with the expansion of immature PBMC subpopulations and the active disease state. Furthermore, an expansion of precursor cells and upregulation of an erythropoiesis gene-expression signature occurred predominantly in individuals with sJIA and anemia. Strikingly, a discrepancy was noted with strong erythropoiesis-related gene expression and expansion of early precursor cells in peripheral circulation and bone marrow but an absence of reticulocytosis.

The etiology and pathogenesis of sJIA are poorly understood. The lack of specific biomarkers makes difficult the diagnosis and differentiation from other febrile conditions, and, therefore, the development of new diagnostic tests is highly desirable. The availability of whole-genome gene-expression analysis technology has allowed the discovery of sJIA-specific gene-expression signatures, opening a window into disease pathogenesis and diagnosis [14,21,22].

In a previous study of sJIA, we demonstrated strong PBMC gene-expression signatures that allowed the distinction between patients with new-onset sJIA and healthy controls [14]. Although several signatures were apparent, the most robust gene-expression signature was an overexpressed 67-probe-set erythropoiesis signature consisting of a large number of strongly erythropoiesis-related mRNAs. "Erythropoiesis" gene-expression signatures have been reported by others (for example, by transcriptional profiling during *in vitro *lineage-specific differentiation of bone marrow-derived CD34^+ ^precursor cells) [23]. In addition, the reticulocyte transcriptome derived from human umbilical cord blood reticulocytes and adult reticulocytes contains many of the genes described in our erythropoiesis signature [24]. In the context of disease and in the peripheral blood, however, reports of erythropoiesis-specific gene-expression signatures have been scarce. The underexpression of erythropoiesis-specific signatures has been reported predominantly [25,26]. Chua *et al. *[25] demonstrated underexpression of an 11-gene erythropoiesis cluster in anemia of chronic renal allograft rejection [25]; of these 11 underexpressed genes, four are found overexpressed within our 67-gene erythropoiesis signature. Ebert *et al. *[26] demonstrated underexpression of a 47-gene erythropoiesis signature in patients with lenalidomide-responsive myelodysplastic syndrome; of those 47 underexpressed genes, 16 are found overexpressed within our 67-gene erythropoiesis signature. Therefore, the presence of a "negative" gene-expression signature in the disease state indicates that the corresponding genes likely are expressed in the healthy state. To our knowledge, overexpression or upregulation of a similar signature was not reported in the literature before our initial description. Notably, in gene-expression studies of sickle cell anemia, which is characterized by severe hemolysis and subsequent expanded erythropoiesis, little to no overlap is found with our gene-expression signature [27].

Our data suggest that the origin of the erythropoiesis-related gene-expression signature may lie within immature and precursor cell subpopulations, most likely CD71^+^, based on the overlap of the signature with the gene-expression pattern observed in isolated CD71^+ ^cells. One flaw of our study is that the study was not designed *a priori *to investigate erythrocyte precursors directly (by flow cytometry) and that some observations were made *a posteriori*. Therefore, more-detailed phenotyping of erythrocyte precursors should be considered in future studies to prove the suggested link. Another strong signature, the "innate immune response cluster," also correlated strongly with the immature cell populations, suggesting that they may have a central role in the pathogenesis of sJIA. The expansion of these cell populations and upregulation of the erythropoiesis-related signature was found most prominently in patients with sJIA and anemia, and it was not seen in patients with other types of JIA and anemia. The signature therefore appears to be a characteristic feature of patients with sJIA. Replication in an independent cohort is the gold standard to validate findings. Our data were confirmed by a non-overlapping cohort previously published by Allantaz *et al. *[21] that included patients with other febrile diseases such as bacterial infections, SLE, and PAPA syndrome. The analysis suggests that the erythropoiesis signature is rather specific for the presence of active sJIA (fever present), whereas it was not present in patients with inactive sJIA (no fever and no arthritis). Of note, a significant number of patients with bacterial infections also overexpressed the erythropoiesis signature, pointing toward a common pathogenic mechanism responsible for the occurrence of the signature.

An important discrepancy with strong erythropoiesis-related gene expression and expansion of precursor cells is found in the context of severe anemia, but conversely, an absence of reticulocytosis, consistent with ineffective erythropoiesis. Ineffective erythropoiesis is the consequence of the premature destruction of erythrocyte precursors either within the bone marrow or shortly after they reach the peripheral blood [28]. According to Cazzola *et al. *[16], the phenotype of the severe anemia in sJIA is characterized by microcytosis, hypoferremia, hyperferritinemia, normal adjusted serum erythropoietin levels, and normal, unadjusted erythroid blast-forming unit levels, which the authors attribute to high circulating levels of interleukin-6 (IL-6). IL-6 is markedly increased in sJIA [29,30], and it has a prominent effect on erythropoiesis [31] by inducing hepatic expression of hepcidin and subsequent decreased intestinal absorption of iron and by inducing ferritin expression in monocytes/macrophages, causing iron sequestration. The overall effect of these actions is a markedly diminished delivery of iron to the proliferating erythrocyte precursor pool. Impaired iron delivery could potentially explain the divergence between the presence of a strong erythropoiesis expression signature and the absence of reticulocytosis as a "maturation block" may occur. In addition, IL-6 has a strong stimulatory effect on other aspects of hematopoiesis [32,33].This suggests that IL-6 may contribute to the expansion of CD34^+ ^cells and the erythropoiesis signature in active sJIA. However, the absence of strong correlation between serum levels of IL-6 and the strength of the signature in our cohort suggests that other factors contribute to the development of this phenomenon. The presence of increased ineffective erythropoiesis has previously been reported in patients with adult rheumatoid arthritis and anemia [28,34,35]. It is possible that the severe anemia observed in sJIA has similar underlying pathogenic mechanisms, but detailed erythrokinetic studies have not been performed in sJIA.

Another striking feature is the similarity between sJIA/MAS, FHLH, and bacterial infection/sepsis in many aspects, with findings of hypercytokinemia, expansion of cytokine-driven macrophages, hemophagocytosis, and an increase in sCD163 levels [36-38]. These similarities suggest a pathogenetic link between these conditions [39] and may suggest that hemophagocytosis plays an important role in sJIA as well. This view is supported by the fact that 30% to 50% of patients with new-onset sJIA have evidence for subclinical hemophagocytosis [7,8]. The close clustering of sJIA, sJIA/MAS, and FHLH patients by using the erythropoiesis signature and the evidence for overexpression of the erythropoiesis signature in some patients with bacterial infection further support the hypothesis of a shared pathogenic mechanism that may include hypercytokinemia, hemophagocytosis, ineffective erythropoiesis, and expansion of immature precursor cells.

## Conclusions

In summary, our findings suggest that the gene-expression pattern seen in active sJIA may be reversible and may be the result of a shared common pathway that can also be seen in FHLH, infection, and SLE. In these conditions, profound, prolonged hypercytokinemia and the resulting hemophagocytosis may lead to ineffective erythropoiesis and the expansion of immature PBMC subpopulations. Furthermore, in the future, gene-expression profiling of a limited set of probes such as that contained in the erythropoiesis signature may be clinically useful for diagnostic purposes and to monitor treatment responses.

## Abbreviations

HLH: hemophagocytic lymphohistiocytosis; JIA: juvenile idiopathic arthritis; MAS: macrophage-activation syndrome; non-sJIA: JIA other than systemic juvenile idiopathic arthritis; PBMCs: peripheral blood mononuclear cells; sJIA: systemic juvenile idiopathic arthritis.

## Competing interests

The authors declare that they have no competing interests.

## Authors' contributions

CH performed the statistical analyses and drafted the manuscript. NF carried out the RNA isolation, some of the flow cytometry, and participated in the statistical analysis. ST, TG, SD, RC, and DG participated in the design of the experiments. MB and AG participated in the design of the experiments, the statistical analyses, and the drafting of the manuscript. JM interpreted the bone marrow biopsy findings and provided images. GLS performed the cytokine analysis. BA participated in the statistical analysis and interpretation. All authors read and approved the final manuscript.

## Supplementary Material

Additional file 1 **The 67 gene erythropoiesis signature**. The file contains a list of the 67 probe sets that are upregulated in patients with sJIA when compared with healthy controls, as reported by Fall *et al. *[14]. Click here for file

Additional file 2 **Clustering of gene-expression profiles from purified cells by using the erythropoiesis signature**. The file contains a heat map showing clustering of RNA samples from purified cell populations. Expression levels of genes related to the 67 probe sets of the erythropoiesis signature (only the 49 that also are found on the U133A) were used to cluster samples from purified cell populations. Additional cell types have been included (bone marrow, whole blood, thymus, tonsil, lymph node, and appendix). The bright band of red on the right represents expression of genes from CD71^+ ^(right-most samples), bone marrow, and CD105^+ ^cells. This is consistent with the overlap of probe sets indicated in Table 4. Each column represents one sample, with the identification of the sample at the bottom. Each row represents the expression of one probe set, with the probe-set IDs and gene symbols shown on the right side of the figure. Colors indicate increased (red) or decreased (blue) expression relative to the median (black) of all samples. Click here for file

Additional file 3 **Differentially expressed probe sets between patients with sJIA with anemia and non-sJIA patients with anemia**. The file contains a list of 671 probe sets in four different clusters that were differentially expressed when comparing patients with sJIA with anemia and non-sJIA patients with anemia. The four clusters are indicated in Figure 2. Click here for file

Additional file 4 **Receiver-operating characteristics (ROC) curve analysis by using the erythropoiesis index in a published cohort of patients with sJIA and other inflammatory conditions**. The file contains figures showing (a) receiver-operating characteristics (ROC) curve analysis, comparing active sJIA (with fever) with bacterial infection and (b) ROC curve analysis, active sJIA (with fever) versus inactive sJIA (no fever). Gene-expression data published by Allantaz *et al. *[21] were retrieved from the Gene Expression Omnibus (GEO) database. Forty-nine probe sets were identical between the Affymetrix U133A (used by Allantaz *et al*.) and the U133 Plus 2.0 arrays (used by our group) and part of the erythropoiesis signature. The geometric mean of the linear expression values of these 49 probe sets was calculated for the individual samples, and the mean for the corresponding groups was calculated. AUC, area under the curve. Click here for file
