# Supplementary material for: Immature cell populations and an erythropoiesis gene-expression signature in systemic juvenile idiopathic arthritis: implications for pathogenesis
Source: Arthritis Res Ther. 2010 Jun 24;12(3):R123. doi: 10.1186/ar3061 (PMC2911917; doi:10.1186/ar3061)
Supplement: Additional file 1 — The 67 gene erythropoiesis signature. The file contains a list of the 67 probe sets that are upregulated in patients with sJIA when compared with healthy controls, as reported by Fall et al. [14]. [file ar3061-S1.DOC]

**Additional file 1:** The 67 gene erythropoiesis signature, up-regulated in patients with sJIA when compared to healthy controls from Fall et al. (14)

| **Probe Set ID** | **Gene Symbol** | **Gene Title** |
| --- | --- | --- |
| 237299_at | --- | --- |
| 239210_at | --- | --- |
| 217748_at | ADIPOR1 | adiponectin receptor 1 |
| 211560_s_at | ALAS2 | aminolevulinate, delta-, synthase 2 |
| 223266_at | ALS2CR2 | amyotrophic lateral sclerosis 2 (juvenile) chromosome region, candidate 2 |
| 209369_at | ANXA3 | annexin A3 |
| 212312_at | BCL2L1 | BCL2-like 1 |
| 221479_s_at | BNIP3L | BCL2/adenovirus E1B 19kDa interacting protein 3-like |
| 203502_at | BPGM | 2,3-bisphosphoglycerate mutase |
| 214273_x_at | C16orf35 | chromosome 16 open reading frame 35 |
| 221764_at | C19orf22 | chromosome 19 open reading frame 22 |
| 55705_at | C19orf22 | chromosome 19 open reading frame 22 |
| 224690_at | C20orf108 | chromosome 20 open reading frame 108 |
| 205950_s_at | CA1 | carbonic anhydrase I |
| 212540_at | CDC34 | cell division cycle 34 homolog (S. cerevisiae) |
| 209498_at | CEACAM1 | carcinoembryonic antigen-related cell adhesion molecule 1 (biliary glycoprotein) |
| 239205_s_at | CR1 /// CR1L | complement component (3b/4b) receptor 1 (Knops blood group) /// complement component (3b/4b) receptor 1-like |
| 201161_s_at | CSDA | cold shock domain protein A |
| 228361_at | E2F2 | E2F transcription factor 2 |
| 214446_at | ELL2 | elongation factor, RNA polymerase II, 2 |
| 210746_s_at | EPB42 | erythrocyte membrane protein band 4.2 |
| 204505_s_at | EPB49 | erythrocyte membrane protein band 4.9 (dematin) |
| 219672_at | ERAF | erythroid associated factor |
| 1556283_s_at | FGFR1OP2 | FGFR1 oncogene partner 2 |
| 226599_at | FHDC1 | FH2 domain containing 1 |
| 208255_s_at | FKBP8 | FK506 binding protein 8, 38kDa |
| 221932_s_at | GLRX5 | glutaredoxin 5 |
| 200648_s_at | GLUL | glutamate-ammonia ligase (glutamine synthetase) |
| 204187_at | GMPR | guanosine monophosphate reductase |
| 201912_s_at | GSPT1 | G1 to S phase transition 1 |
| 211821_x_at | GYPA | glycophorin A (MNS blood group) |
| 202947_s_at | GYPC | glycophorin C (Gerbich blood group) |
| 206834_at | HBB /// HBD | hemoglobin, beta /// hemoglobin, delta |
| 213515_x_at | HBG1 /// HBG2 | hemoglobin, gamma A /// hemoglobin, gamma G |
| 240336_at | HBM | hemoglobin, mu |
| 220807_at | HBQ1 | hemoglobin, theta 1 |
| 209398_at | HIST1H1C | histone cluster 1, H1c |
| 225800_at | JAZF1 | JAZF zinc finger 1 |
| 210504_at | KLF1 | Kruppel-like factor 1 (erythroid) |
| 231982_at | LOC284422 | similar to HSPC323 |
| 209845_at | MKRN1 | makorin ring finger protein 1 |
| 202974_at | MPP1 | membrane protein, palmitoylated 1, 55kDa |
| 210395_x_at | MYL4 | myosin, light chain 4, alkali; atrial, embryonic |
| 212445_s_at | NEDD4L | neural precursor cell expressed, developmentally down-regulated 4-like |
| 206302_s_at | NUDT4 /// NUDT4P1 | nudix (nucleoside diphosphate linked moiety X)-type motif 4 /// nudix (nucleoside diphosphate linked moiety X)-type motif 4 pseudogene 1 |
| 241881_at | OR2W3 | olfactory receptor, family 2, subfamily W, member 3 |
| 223432_at | OSBP2 | oxysterol binding protein 2 |
| 227935_s_at | PCGF5 | polycomb group ring finger 5 |
| 209018_s_at | PINK1 | PTEN induced putative kinase 1 |
| 218644_at | PLEK2 | pleckstrin 2 |
| 202129_s_at | RIOK3 | RIO kinase 3 (yeast) |
| 207801_s_at | RNF10 | ring finger protein 10 |
| 202083_s_at | SEC14L1 | SEC14-like 1 (S. cerevisiae) |
| 214433_s_at | SELENBP1 | selenium binding protein 1 |
| 235683_at | SESN3 | sestrin 3 |
| 209339_at | SIAH2 | seven in absentia homolog 2 (Drosophila) |
| 205896_at | SLC22A4 | solute carrier family 22 (organic cation/ergothioneine transporter), member 4 |
| 222529_at | SLC25A37 | solute carrier family 25, member 37 |
| 223649_s_at | SLC25A39 | solute carrier family 25, member 39 |
| 205592_at | SLC4A1 | solute carrier family 4, anion exchanger, member 1 (erythrocyte membrane protein band 3, Diego blood group) |
| 213843_x_at | SLC6A8 | solute carrier family 6 (neurotransmitter transporter, creatine), member 8 |
| 211546_x_at | SNCA | synuclein, alpha (non A4 component of amyloid precursor) |
| 213096_at | TMCC2 | transmembrane and coiled-coil domain family 2 |
| 203662_s_at | TMOD1 | tropomodulin 1 |
| 221627_at | TRIM10 | tripartite motif-containing 10 |
| 220757_s_at | UBXN6 | UBX domain protein 6 |
| 227309_at | YOD1 | YOD1 OTU deubiquinating enzyme 1 homolog (S. cerevisiae) |
