# Supplementary material for: Immature cell populations and an erythropoiesis gene-expression signature in systemic juvenile idiopathic arthritis: implications for pathogenesis
Source: Arthritis Res Ther. 2010 Jun 24;12(3):R123. doi: 10.1186/ar3061 (PMC2911917; doi:10.1186/ar3061)
Supplement: Additional file 2 — Clustering of gene-expression profiles from purified cells by using the erythropoiesis signature. The file contains a heat map showing clustering of RNA samples from purified cell populations. Expression levels of genes related to the 67 probe sets of the erythropoiesis signature (only the 49 that also are found on the U133A) were used to cluster samples from purified cell populations. Additional cell types have been included (bone marrow, whole blood, thymus, tonsil, lymph node, and appendix). The bright band of red on the right represents expression of genes from CD71+ (right-most samples), bone marrow, and CD105+ cells. This is consistent with the overlap of probe sets indicated in Table 4. Each column represents one sample, with the identification of the sample at the bottom. Each row represents the expression of one probe set, with the probe-set IDs and gene symbols shown on the right side of the figure. Colors indicate increased (red) or decreased (blue) expression relative to the median (black) of all samples. [file ar3061-S2.DOC]

Additional file 2: Clustering of gene expression profiles from purified cells.


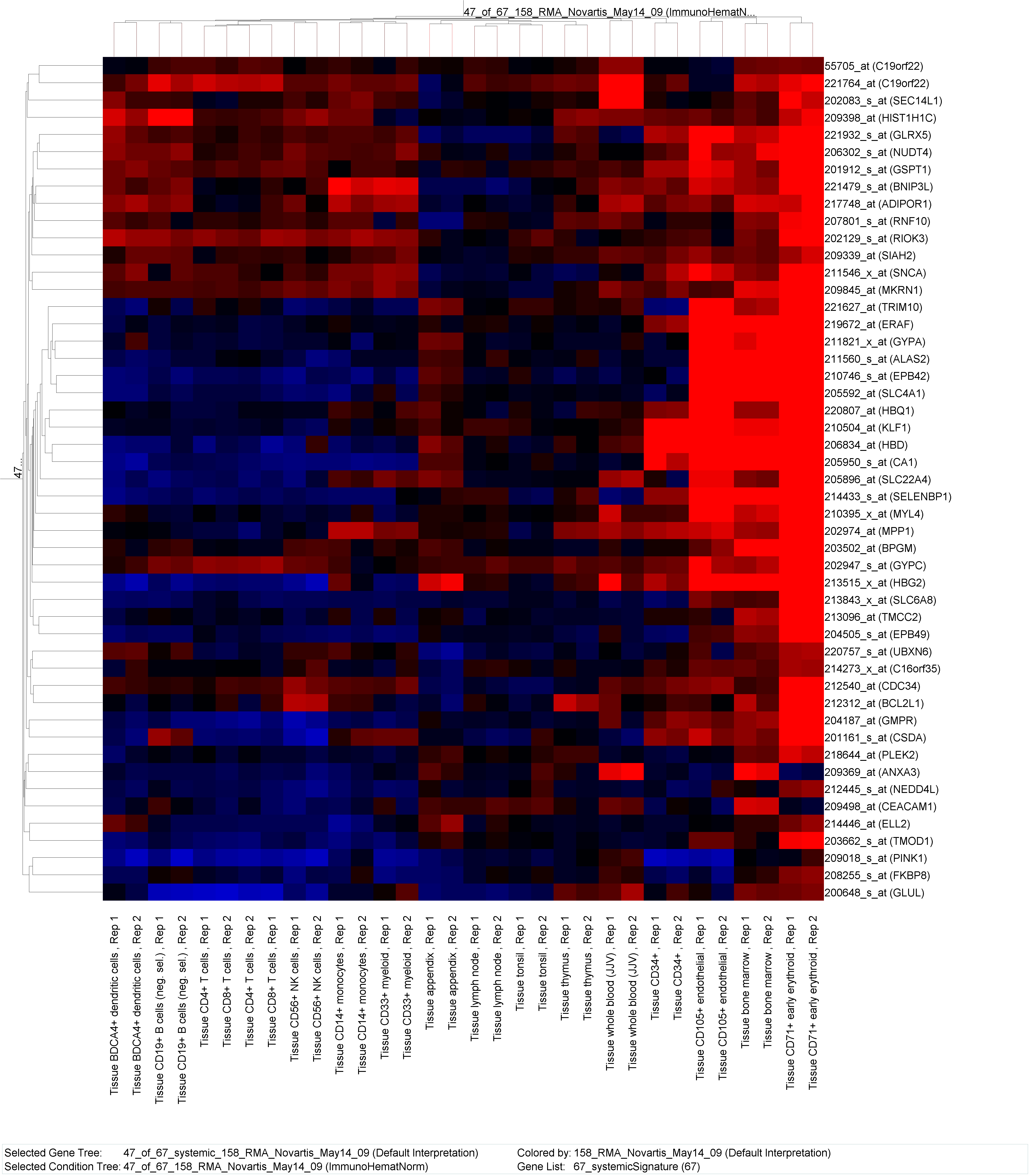


Expression levels of genes related to the 67 probe sets of the erythropoiesis signature (only the 49 that are also found on the U133A) were used to cluster samples from purified cell populations. In this figure, additional cell types have been included (bone marrow, whole blood, thymus, tonsil, lymph node, and appendix). The bright band of red on the right represent expression of genes from CD71+ (right most samples), bone marrow, and CD105+ cells. This is consistent with the overlap of probe sets indicated in Table 4. Each column represents one sample with the identification of the sample at the bottom. Each row represents expression of 1 probe set with the probe set IDs and gene symbols shown on the right hand side of the figure. Colors indicate increased (red) or decreased (blue) expression relative to the median (black) of all samples.
