# Supplementary material for: Immature cell populations and an erythropoiesis gene-expression signature in systemic juvenile idiopathic arthritis: implications for pathogenesis
Source: Arthritis Res Ther. 2010 Jun 24;12(3):R123. doi: 10.1186/ar3061 (PMC2911917; doi:10.1186/ar3061)
Supplement: Additional file 3 — Differentially expressed probe sets between patients with sJIA with anemia and non-sJIA patients with anemia. The file contains a list of 671 probe sets in four different clusters that were differentially expressed when comparing patients with sJIA with anemia and non-sJIA patients with anemia. The four clusters are indicated in Figure 2. [file ar3061-S3.DOC]

Additional file 3: Differentially expressed probe sets when comparing patients with sJIA with anemia to patients with non-sJIA with anemia. 671 probe sets were differentially expressed and 4 separate clusters (I-IV) emerged (see Figure 2.

| Cluster I |  |  |  |
| --- | --- | --- | --- |
|  |  |  |  |
| **Affymetrix ID** | **Gene name** | **fold change** | **p-value** |
| 230820_at |  | 0.81 | 0.04 |
| 205596_s_at | SMURF2 | 0.82 | 0.02 |
| 235556_at | LOC153222 | 0.80 | 0.04 |
| 218972_at | TTC17 | 0.74 | 0.01 |
| 203241_at | UVRAG | 0.76 | 0.04 |
| 209388_at | PAPOLA | 0.80 | 0.04 |
| 229955_at | FBXO3 | 0.77 | 0.04 |
| 236196_at |  | 0.73 | 0.04 |
| 229844_at |  | 0.70 | 0.04 |
| 223287_s_at | FOXP1 | 0.74 | 0.02 |
| 239498_at | MRRF | 0.82 | 0.04 |
| 229356_x_at | INOC1 | 0.81 | 0.03 |
| 210396_s_at | LOC440354 | 0.77 | 0.02 |
| 227493_s_at | KIAA1143 | 0.78 | 0.02 |
| 204977_at | DDX10 | 0.71 | 0.01 |
| 1565949_x_at | CHML | 0.82 | 0.04 |
| 202113_s_at | SNX2 | 0.75 | 0.02 |
| 202548_s_at | ARHGEF7 | 0.80 | 0.04 |
| 204057_at | IRF8 | 0.79 | 0.03 |
| 202079_s_at | TRAK1 | 0.79 | 0.04 |
| 202080_s_at | TRAK1 | 0.73 | 0.03 |
| 226635_at | LOC401504 | 0.77 | 0.04 |
| 229700_at | ZNF738 | 0.74 | 0.02 |
| 218311_at | MAP4K3 | 0.72 | 0.04 |
| 229393_at | L3MBTL3 | 0.71 | 0.01 |
| 1554627_a_at | ASCC1 | 0.75 | 0.04 |
| 223135_s_at | BBX | 0.77 | 0.03 |
| 201607_at | PWP1 | 0.76 | 0.03 |
| 213239_at | C13orf24 | 0.75 | 0.02 |
| 203313_s_at | TGIF | 0.68 | 0.02 |
| 203431_s_at | RICS | 0.64 | 0.02 |
| 237143_at |  | 0.67 | 0.04 |
| 1555562_a_at | ZCCHC7 | 0.69 | 0.04 |
| 218696_at | EIF2AK3 | 0.64 | 0.02 |
| 229116_at |  | 0.69 | 0.02 |
| 226959_at |  | 0.67 | 0.02 |
| 204995_at | CDK5R1 | 0.67 | 0.03 |
| 226016_at | CD47 | 0.89 | 0.05 |
| 212455_at | YTHDC1 | 0.90 | 0.02 |
| 225216_at | CXorf39 | 0.84 | 0.01 |
| 228991_at |  | 0.85 | 0.04 |
| 222728_s_at | JOSD3 | 0.82 | 0.03 |
| 200912_s_at | EIF4A2 | 0.87 | 0.02 |
| 209377_s_at | HMGN3 | 0.83 | 0.02 |
| 201368_at | ZFP36L2 | 0.85 | 0.01 |
| 226215_s_at | FBXL10 | 0.82 | 0.01 |
| 201165_s_at | PUM1 | 0.88 | 0.05 |
| 225204_at | PPTC7 | 0.86 | 0.02 |
| 225213_at | PPTC7 | 0.81 | 0.01 |
| 201994_at | MORF4L2 | 0.89 | 0.04 |
| 201010_s_at | TXNIP | 0.82 | 0.01 |
| 201327_s_at | CCT6A | 0.86 | 0.02 |
| 201257_x_at | RPS3A | 0.92 | 0.04 |
| 209134_s_at | RPS6 | 0.92 | 0.04 |
| 212042_x_at | RPL7 | 0.90 | 0.01 |
| 200717_x_at | RPL7 | 0.92 | 0.04 |
| 208635_x_at | NACA | 0.93 | 0.03 |
| 200735_x_at | NACA | 0.93 | 0.02 |
| 200888_s_at | RPL23 | 0.96 | 0.04 |
| 213477_x_at | EEF1A1 | 0.94 | 0.03 |
| 213583_x_at | EEF1A1 | 0.93 | 0.02 |
| 200847_s_at | TMEM66 | 0.92 | 0.02 |
| 200926_at | RPS23 | 0.93 | 0.03 |
| 214143_x_at | RPL24 | 0.94 | 0.03 |
| 201575_at | SNW1 | 0.91 | 0.04 |
| 201880_at | ARIH1 | 0.89 | 0.03 |
| 222099_s_at | LSM14A | 0.91 | 0.03 |
| 206158_s_at | CNBP | 0.90 | 0.03 |
| 201857_at | ZFR | 0.88 | 0.03 |
| 209685_s_at | PRKCB1 | 0.90 | 0.03 |
| 222729_at | FBXW7 | 0.87 | 0.04 |
| 215424_s_at | SNW1 | 0.87 | 0.04 |
| 217900_at | IARS2 | 0.87 | 0.03 |
| 234969_s_at | EPC1 | 0.83 | 0.01 |
| 201816_s_at | GBAS | 0.83 | 0.01 |
| 201112_s_at | CSE1L | 0.85 | 0.04 |
| 211988_at | SMARCE1 | 0.84 | 0.02 |
| 218973_at | EFTUD1 | 0.81 | 0.02 |
| 212106_at | UBXD8 | 0.80 | 0.03 |
| 232008_s_at | BBX | 0.80 | 0.04 |
| 210946_at | PPAP2A | 0.75 | 0.01 |
| 227447_at | SKIV2L2 | 0.83 | 0.01 |
| 226227_x_at | TALDO1 | 0.81 | 0.05 |
| 211955_at | RANBP5 | 0.79 | 0.04 |
| 223177_at | NT5DC1 | 0.80 | 0.04 |
| 209064_x_at | PAIP1 | 0.81 | 0.05 |
| 201606_s_at | PWP1 | 0.80 | 0.04 |
| 218250_s_at | CNOT7 | 0.86 | 0.04 |
| 227630_at |  | 0.82 | 0.02 |
| 202088_at | SLC39A6 | 0.82 | 0.04 |
| 208737_at | ATP6V1G1 | 0.83 | 0.04 |
| 201008_s_at | TXNIP | 0.79 | 0.02 |
| 201009_s_at | TXNIP | 0.77 | 0.00 |
| 226796_at | LOC116236 | 0.76 | 0.04 |
| 221842_s_at | ZNF131 | 0.80 | 0.02 |
| 214741_at | ZNF131 | 0.75 | 0.02 |
| 209199_s_at | MEF2C | 0.77 | 0.04 |
| 225174_at | DNAJC10 | 0.78 | 0.03 |
| 224837_at | FOXP1 | 0.76 | 0.03 |
| 226496_at | ZCCHC7 | 0.74 | 0.02 |
| 229146_at | C7orf31 | 0.67 | 0.01 |
| 229588_at | DNAJC10 | 0.71 | 0.02 |
| 226382_at | LOC283070 | 0.69 | 0.02 |
| 204119_s_at | ADK | 0.69 | 0.02 |
| 217833_at | SYNCRIP | 0.82 | 0.05 |
| 224755_at |  | 0.81 | 0.05 |
| 212893_at | ZZZ3 | 0.79 | 0.03 |
| 213704_at | RABGGTB | 0.76 | 0.04 |
| 203341_at | CEBPZ | 0.82 | 0.04 |
| 227861_at | TMEM161B | 0.78 | 0.05 |
| 218769_s_at | ANKRA2 | 0.79 | 0.04 |
| 229317_at |  | 0.75 | 0.04 |
| 219443_at | TASP1 | 0.76 | 0.04 |
| 212462_at | MYST4 | 0.80 | 0.04 |
| 215716_s_at | ATP2B1 | 0.75 | 0.03 |
| 225470_at | NUP35 | 0.79 | 0.02 |
| 225768_at | NR1D2 | 0.78 | 0.04 |
| 228604_at |  | 0.84 | 0.04 |
| 226449_at | CCDC100 | 0.79 | 0.02 |
| 225417_at | EPC1 | 0.82 | 0.02 |
| 235980_at |  | 0.78 | 0.05 |
| 243868_at |  | 0.75 | 0.02 |
| 224838_at | FOXP1 | 0.87 | 0.05 |
| 203250_at | RBM16 | 0.88 | 0.04 |
| 209481_at | SNRK | 0.84 | 0.02 |
| 201166_s_at | PUM1 | 0.91 | 0.04 |
| 217779_s_at | PNRC2 | 0.91 | 0.02 |
| 208766_s_at | HNRPR | 0.91 | 0.01 |
| 200626_s_at | MATR3 | 0.89 | 0.03 |
| 218499_at | RP6-213H19.1 | 0.88 | 0.04 |
| 201916_s_at | SEC63 | 0.83 | 0.02 |
| 201595_s_at | LEREPO4 | 0.85 | 0.01 |
| 224726_at | MIB1 | 0.84 | 0.04 |
| 226148_at | BTBD15 | 0.85 | 0.03 |
| 218236_s_at | PRKD3 | 0.80 | 0.04 |
| 222565_s_at | PRKD3 | 0.80 | 0.02 |
| 227767_at | CSNK1G3 | 0.80 | 0.03 |
| 213725_x_at | LOC283824 | 0.75 | 0.01 |
| 227239_at | DRCTNNB1A | 0.79 | 0.04 |
| 204635_at | RPS6KA5 | 0.77 | 0.02 |
| 218191_s_at | LMBRD1 | 0.83 | 0.03 |
| 203182_s_at | SRPK2 | 0.81 | 0.02 |
| 203020_at | RABGAP1L | 0.81 | 0.03 |
| 242669_at | UFM1 | 0.71 | 0.03 |
| 212239_at | PIK3R1 | 0.81 | 0.05 |
| 225845_at | BTBD15 | 0.78 | 0.02 |
| 224734_at | HMGB1 | 0.79 | 0.01 |
| 209112_at | CDKN1B | 0.77 | 0.01 |
| 218919_at | ZFAND1 | 0.76 | 0.01 |
| 239101_at | ITCH | 0.76 | 0.02 |
| 204244_s_at | DBF4 | 0.78 | 0.05 |
| 218462_at | BXDC5 | 0.85 | 0.04 |
| 213080_x_at | RPL5 | 0.91 | 0.04 |
| 200034_s_at | RPL6 | 0.87 | 0.02 |
| 200063_s_at | NPM1 | 0.87 | 0.02 |
| 211971_s_at | LRPPRC | 0.83 | 0.01 |
| 207483_s_at | CAND1 | 0.84 | 0.02 |
| 210766_s_at | CSE1L | 0.83 | 0.02 |
| 226680_at | IKZF5 | 0.83 | 0.03 |
| 201456_s_at | BUB3 | 0.82 | 0.04 |
| 209092_s_at | C17orf25 | 0.83 | 0.03 |
| 214173_x_at | C19orf2 | 0.77 | 0.02 |
| 206555_s_at | THUMPD1 | 0.83 | 0.03 |
| 200937_s_at | RPL5 | 0.84 | 0.03 |
| 200624_s_at | MATR3 | 0.83 | 0.01 |
| 202165_at | PPP1R2 | 0.81 | 0.03 |
| 239133_at |  | 0.81 | 0.01 |
| 228569_at | PAPOLA | 0.80 | 0.03 |
| 239897_at | BCLAF1 | 0.80 | 0.02 |
| 222947_at | ZNF224 | 0.83 | 0.03 |
| 1554329_x_at | STXBP4 | 0.79 | 0.02 |
| 244032_at |  | 0.77 | 0.04 |
| 238662_at | ATPBD4 | 0.79 | 0.03 |
| 1569472_s_at | TTC3 | 0.69 | 0.01 |
| 207543_s_at | P4HA1 | 0.78 | 0.05 |
| 230264_s_at | AP1S2 | 0.81 | 0.03 |
| 230413_s_at | AP1S2 | 0.80 | 0.03 |
| 203299_s_at | AP1S2 | 0.72 | 0.01 |
| 239034_at | CXorf24 | 0.78 | 0.04 |
| 227034_at | ANKRD57 | 0.71 | 0.04 |
| 205051_s_at | KIT | 0.71 | 0.02 |
| 223686_at | TPK1 | 0.70 | 0.04 |
| 221218_s_at | TPK1 | 0.73 | 0.03 |
| 224392_s_at | OPN3 | 0.68 | 0.02 |
| 219032_x_at | OPN3 | 0.73 | 0.02 |
| 1557984_s_at | FLJ21908 | 0.68 | 0.05 |
| 235645_at | ESCO1 | 0.74 | 0.04 |
| 1564962_at | ZNF92 | 0.66 | 0.01 |
| 1565389_s_at | GRM5 | 0.77 | 0.04 |
| 229462_at |  | 0.80 | 0.02 |
| 219738_s_at | PCDH9 | 0.70 | 0.02 |
| 223535_at | NUDT12 | 0.70 | 0.02 |
| 233609_at | PTPRK | 0.61 | 0.02 |
| 236699_at | MBNL2 | 0.51 | 0.01 |
| 239938_x_at | MEF2C | 0.58 | 0.02 |
| 227198_at | AFF3 | 0.58 | 0.04 |
| 226550_at |  | 0.59 | 0.04 |
| 217979_at | TSPAN13 | 0.65 | 0.04 |
| 227173_s_at | BACH2 | 0.60 | 0.02 |
| 226350_at | CHML | 0.64 | 0.02 |
| 1565951_s_at | CHML | 0.55 | 0.01 |
| 222520_s_at | IFT57 | 0.55 | 0.01 |
| 226844_at | MOBKL2B | 0.59 | 0.04 |
| 213172_at | TTC9 | 0.59 | 0.04 |
| 244463_at |  | 0.59 | 0.04 |
| 217820_s_at | ENAH | 0.58 | 0.04 |
| 235650_at | FLJ23834 | 0.62 | 0.02 |
| 228976_at | ICOSLG | 0.60 | 0.04 |
| 243364_at | AUTS2 | 0.62 | 0.01 |
| 1553856_s_at | P2RY10 | 0.63 | 0.03 |
| 214615_at | P2RY10 | 0.58 | 0.01 |
| 1558378_a_at | C14orf78 | 0.59 | 0.03 |
| 211656_x_at | HLA-DQB1 | 0.62 | 0.05 |
| 210982_s_at | HLA-DRA | 0.68 | 0.05 |
| 228555_at | CAMK2D | 0.68 | 0.04 |
| 211990_at | HLA-DPA1 | 0.64 | 0.04 |
| 213537_at | HLA-DPA1 | 0.52 | 0.03 |
| 206100_at | CPM | 0.55 | 0.03 |
| 203543_s_at | KLF9 | 0.67 | 0.04 |
| 228005_at | ZXDB | 0.70 | 0.04 |
| 201417_at | SOX4 | 0.65 | 0.03 |
| 1566514_at | CWF19L2 | 0.69 | 0.04 |
| 202393_s_at | KLF10 | 0.64 | 0.04 |
| 218486_at | KLF11 | 0.64 | 0.04 |
| 236105_at |  | 0.62 | 0.04 |
| 235780_at | PRKACB | 0.63 | 0.04 |
| 1554264_at | CKAP2 | 0.63 | 0.04 |
| 244443_at | LOC440309 | 0.67 | 0.04 |
| 236778_at | ATRX | 0.57 | 0.02 |
| 204702_s_at | NFE2L3 | 0.74 | 0.05 |
| 205327_s_at | ACVR2A | 0.72 | 0.03 |
| 228933_at | NHS | 0.63 | 0.03 |
| 223331_s_at | DDX20 | 0.74 | 0.05 |
| 235369_at | C14orf28 | 0.75 | 0.04 |
| 206648_at | ZNF571 | 0.68 | 0.02 |
| 1555500_s_at | SLC2A4RG | 0.73 | 0.04 |
| 227329_at | BTBD4 | 0.71 | 0.02 |
| 235635_at | ARHGAP5 | 0.68 | 0.04 |
| 229705_at |  | 0.67 | 0.02 |
| 225147_at | PSCD3 | 0.64 | 0.02 |
| 243951_at | ABCB1 | 0.67 | 0.02 |
| 212599_at | AUTS2 | 0.62 | 0.01 |
| 222258_s_at | SH3BP4 | 0.62 | 0.01 |
| 244447_at | KLF10 | 0.55 | 0.03 |
| 213808_at |  | 0.55 | 0.05 |
| 1565601_at | PCDH9 | 0.48 | 0.05 |
| 219737_s_at | PCDH9 | 0.43 | 0.03 |
| 212094_at | PEG10 | 0.43 | 0.05 |
| 1552742_at | KCNH8 | 0.37 | 0.03 |
| 210222_s_at | RTN1 | 0.54 | 0.04 |
| 203485_at | RTN1 | 0.48 | 0.03 |
| 219093_at | FLJ20701 | 0.42 | 0.02 |
| 231650_s_at | SEZ6L | 0.55 | 0.01 |
| 213609_s_at | SEZ6L | 0.47 | 0.00 |
| 1559910_at | FLJ20701 | 0.40 | 0.04 |
| 219714_s_at | CACNA2D3 | 0.30 | 0.02 |
|  |  |  |  |
| Cluster II |  |  |  |
|  |  |  |  |
| 207384_at | PGLYRP1 | 2.96 | 0.04 |
| 227297_at | ITGA9 | 2.76 | 0.05 |
| 223767_at | GPR84 | 3.26 | 0.04 |
| 211889_x_at | CEACAM1 | 3.18 | 0.03 |
| 211883_x_at | CEACAM1 | 3.74 | 0.02 |
| 229490_s_at |  | 3.40 | 0.01 |
| 204304_s_at | PROM1 | 2.85 | 0.04 |
| 208335_s_at | DARC | 2.62 | 0.05 |
| 221425_s_at | HBLD2 | 2.35 | 0.04 |
| 1554481_a_at | EPB41 | 2.65 | 0.02 |
| 229622_at | FLJ37034 | 2.55 | 0.04 |
| 224051_at |  | 2.39 | 0.04 |
| 210215_at | TFR2 | 2.66 | 0.02 |
| 205390_s_at | ANK1 | 2.25 | 0.04 |
| 201333_s_at | ARHGEF12 | 2.22 | 0.04 |
| 241129_at | ALS2CR2 | 2.52 | 0.02 |
| 205391_x_at | ANK1 | 2.13 | 0.03 |
| 208352_x_at | ANK1 | 2.48 | 0.02 |
| 207087_x_at | ANK1 | 2.31 | 0.01 |
| 222678_s_at | DCUN1D1 | 2.41 | 0.02 |
| 221824_s_at | 8-Mar | 2.34 | 0.03 |
| 216925_s_at | TAL1 | 2.57 | 0.02 |
| 209019_s_at | PINK1 | 2.47 | 0.01 |
| 209018_s_at | PINK1 | 2.77 | 0.01 |
| 212540_at | CDC34 | 2.30 | 0.01 |
| 202201_at | BLVRB | 2.37 | 0.04 |
| 230778_at |  | 2.32 | 0.04 |
| 212473_s_at | MICAL2 | 2.30 | 0.03 |
| 212472_at | MICAL2 | 2.53 | 0.03 |
| 217799_x_at | UBE2H | 2.08 | 0.04 |
| 226681_at | UBE2H | 2.07 | 0.03 |
| 204507_s_at | PPP3R1 | 1.93 | 0.04 |
| 232579_at |  | 2.17 | 0.01 |
| 212340_at | YIPF6 | 2.51 | 0.02 |
| 239142_at | LOC317671 | 2.46 | 0.03 |
| 223597_at | ITLN1 | 2.50 | 0.03 |
| 214446_at | ELL2 | 2.18 | 0.03 |
| 1569206_at | TCP11L2 | 2.37 | 0.02 |
| 213319_s_at | CSDA | 2.32 | 0.05 |
| 209807_s_at | NFIX | 2.51 | 0.03 |
| 221764_at | C19orf22 | 2.15 | 0.02 |
| 55705_at | C19orf22 | 2.09 | 0.02 |
| 231548_at | FOXO3A | 2.11 | 0.04 |
| 224889_at | FOXO3A | 2.09 | 0.03 |
| 204131_s_at | FOXO3A | 2.33 | 0.02 |
| 221675_s_at | CHPT1 | 2.12 | 0.04 |
| 202364_at | MXI1 | 2.22 | 0.03 |
| 207667_s_at | MAP2K3 | 2.12 | 0.03 |
| 1557455_s_at | MOSPD1 | 2.24 | 0.04 |
| 200648_s_at | GLUL | 2.16 | 0.05 |
| 217202_s_at | GLUL | 2.33 | 0.04 |
| 201335_s_at | ARHGEF12 | 2.55 | 0.04 |
| 209339_at | SIAH2 | 2.57 | 0.04 |
| 202131_s_at | RIOK3 | 2.56 | 0.02 |
| 202468_s_at | CTNNAL1 | 3.00 | 0.02 |
| 202129_s_at | RIOK3 | 3.16 | 0.01 |
| 202974_at | MPP1 | 2.51 | 0.02 |
| 217748_at | ADIPOR1 | 2.56 | 0.02 |
| 214464_at | CDC42BPA | 2.08 | 0.05 |
| 239458_at | ALS2CR12 | 2.01 | 0.04 |
| 222043_at | CLU | 1.90 | 0.03 |
| 200615_s_at | AP2B1 | 1.60 | 0.04 |
| 202082_s_at | SEC14L1 | 1.67 | 0.04 |
| 226657_at | DKFZp762H185 | 2.04 | 0.02 |
| 200983_x_at | CD59 | 1.85 | 0.04 |
| 222513_s_at | SORBS1 | 2.03 | 0.04 |
| 201061_s_at | STOM | 1.97 | 0.04 |
| 210824_at |  | 2.46 | 0.02 |
| 214428_x_at | C4A | 2.37 | 0.03 |
| 231997_at | LRRC35 | 1.92 | 0.05 |
| 223865_at | SOX6 | 2.01 | 0.03 |
| 242899_at |  | 1.88 | 0.04 |
| 235512_at | CDKL1 | 1.85 | 0.04 |
| 215438_x_at | GSPT1 | 1.83 | 0.04 |
| 209845_at | MKRN1 | 2.20 | 0.03 |
| 211992_at | WNK1 | 1.94 | 0.04 |
| 211993_at | WNK1 | 2.06 | 0.02 |
| 213310_at | EIF2C2 | 2.20 | 0.02 |
| 224186_s_at | RNF123 | 1.59 | 0.05 |
| 215684_s_at | ASCC2 | 1.88 | 0.02 |
| 225074_at | RAB2B | 1.77 | 0.02 |
| 212330_at | TFDP1 | 1.73 | 0.01 |
| 213161_at | C9orf97 | 2.03 | 0.01 |
| 213439_x_at | RPIP8 | 2.02 | 0.02 |
| 225168_at | FRMD4A | 2.15 | 0.01 |
| 227252_at | LRP10 | 1.85 | 0.01 |
| 214530_x_at | EPB41 | 1.98 | 0.04 |
| 206665_s_at | BCL2L1 | 1.89 | 0.04 |
| 229122_x_at |  | 2.07 | 0.02 |
| 218863_s_at | TNS1 | 2.04 | 0.02 |
| 228712_at | WNK1 | 1.75 | 0.04 |
| 208416_s_at | SPTB | 2.04 | 0.02 |
| 1554423_a_at | FBXO7 | 1.80 | 0.02 |
| 201178_at | FBXO7 | 1.79 | 0.01 |
| 208632_at | RNF10 | 1.86 | 0.02 |
| 224891_at | FOXO3A | 1.75 | 0.04 |
| 203966_s_at | PPM1A | 1.79 | 0.02 |
| 231370_at | PPM1A | 1.88 | 0.02 |
| 224905_at | WDR26 | 1.80 | 0.03 |
| 224898_at | WDR26 | 1.90 | 0.03 |
| 1566509_s_at | FBXO9 | 1.77 | 0.02 |
| 224790_at | DDEF1 | 1.94 | 0.02 |
| 226179_at | LOC653778 | 2.37 | 0.00 |
| 211781_x_at |  | 2.13 | 0.02 |
| 211702_s_at | USP32 | 2.08 | 0.01 |
| 36936_at | TSTA3 | 2.10 | 0.04 |
| 223012_at | UBXD1 | 2.25 | 0.03 |
| 200075_s_at | GUK1 | 2.20 | 0.03 |
| 218872_at | TESC | 2.10 | 0.04 |
| 205570_at | PIP5K2A | 2.01 | 0.03 |
| 211475_s_at | BAG1 | 2.17 | 0.03 |
| 202387_at | BAG1 | 2.17 | 0.03 |
| 216951_at | FCGR1A | 2.14 | 0.04 |
| 214511_x_at | FCGR1A | 2.21 | 0.03 |
| 216950_s_at | FCGR1A | 2.20 | 0.03 |
| 202800_at | SLC1A3 | 2.49 | 0.02 |
| 218660_at | DYSF | 2.16 | 0.04 |
| 224707_at | ORF1-FL49 | 2.87 | 0.03 |
| 202912_at | ADM | 2.12 | 0.02 |
| 226726_at | MBOAT2 | 2.51 | 0.02 |
| 209930_s_at | NFE2 | 2.53 | 0.01 |
| 227055_at | METTL7B | 2.69 | 0.02 |
| 219607_s_at | MS4A4A | 2.69 | 0.03 |
| 1555728_a_at | MS4A4A | 2.36 | 0.02 |
| 241981_at | FAM20A | 3.56 | 0.01 |
| 226804_at | FAM20A | 2.75 | 0.01 |
| 242945_at | FAM20A | 3.19 | 0.00 |
| 219919_s_at | SSH3 | 1.31 | 0.05 |
|  |  |  |  |
| Cluster III |  |  |  |
|  |  |  |  |
| 226804_at | FAM20A | 2.75 | 0.01 |
| 242945_at | FAM20A | 3.19 | 0.00 |
| 219919_s_at | SSH3 | 1.31 | 0.05 |
| 239528_at | PROM2 | 1.18 | 0.05 |
| 219744_at | FN3K | 1.19 | 0.02 |
| 232546_at | TP73 | 1.14 | 0.04 |
| 207242_s_at | GRIK1 | 1.14 | 0.03 |
| 240274_at | EPB42 | 1.16 | 0.03 |
| 1552749_a_at | KLC3 | 1.17 | 0.02 |
| 211679_x_at | GABBR2 | 1.15 | 0.01 |
| 1558705_at | ATOH8 | 1.20 | 0.03 |
| 234722_x_at | OBP2B | 1.19 | 0.05 |
| 201187_s_at | ITPR3 | 1.20 | 0.04 |
| 1561384_a_at | LOC284661 | 1.16 | 0.03 |
| 201261_x_at | BGN | 1.22 | 0.02 |
| 232813_s_at | GOLGA | 1.16 | 0.04 |
| 1554459_s_at | CFHR3 | 1.12 | 0.03 |
| 217676_at |  | 1.15 | 0.03 |
| 207529_at | DEFA5 | 1.14 | 0.03 |
| 1561545_at |  | 1.12 | 0.04 |
| 205813_s_at | MAT1A | 1.14 | 0.02 |
| 1553053_at | C14orf48 | 1.15 | 0.03 |
| 238163_at |  | 1.18 | 0.02 |
| 231510_at | GLI2 | 1.20 | 0.04 |
| 207372_s_at | ENTPD2 | 1.19 | 0.04 |
| 241056_at |  | 1.24 | 0.01 |
| 230406_at |  | 1.17 | 0.04 |
| 1556015_a_at | MESP2 | 1.21 | 0.04 |
| 215119_at | RP11-54H7.1 | 1.19 | 0.03 |
| 1554554_at | CCDC57 | 1.14 | 0.04 |
| 243850_at |  | 1.14 | 0.02 |
| 231781_s_at | LRRC2 | 1.16 | 0.03 |
| 212987_at | FBXO9 | 1.16 | 0.02 |
| 240488_at |  | 1.16 | 0.01 |
| 216730_at |  | 1.22 | 0.03 |
| 232147_at | BTBD12 | 1.22 | 0.04 |
| 203193_at | ESRRA | 1.19 | 0.03 |
| 1566927_at | C21orf104 | 1.24 | 0.03 |
| 207542_s_at | AQP1 | 1.25 | 0.02 |
| 219331_s_at | KLHDC8A | 1.22 | 0.05 |
| 210151_s_at | DYRK3 | 1.28 | 0.02 |
| 218194_at | REXO2 | 1.23 | 0.03 |
| 236981_at |  | 1.25 | 0.01 |
| 1553169_at | C20orf75 | 1.26 | 0.02 |
| 217683_at | HBE1 | 1.25 | 0.01 |
| 219786_at | MTL5 | 1.27 | 0.05 |
| 211713_x_at | KIAA0101 | 1.27 | 0.04 |
| 241177_at |  | 1.32 | 0.05 |
| 227677_at | JAK3 | 1.28 | 0.02 |
| 212342_at | YIPF6 | 1.34 | 0.03 |
| 237367_x_at | CFLAR | 1.30 | 0.02 |
| 217836_s_at | YY1AP1 | 1.35 | 0.03 |
| 202568_s_at | MARK3 | 1.38 | 0.02 |
| 210638_s_at | FBXO9 | 1.46 | 0.02 |
| 217572_at |  | 1.30 | 0.04 |
| 206077_at | KEL | 1.36 | 0.04 |
| 236218_at | PHOSPHO1 | 1.46 | 0.02 |
| 236056_s_at |  | 1.40 | 0.04 |
| 220778_x_at | SEMA6B | 1.26 | 0.04 |
| 224182_x_at | SEMA6B | 1.32 | 0.02 |
| 228939_at | OAF | 1.28 | 0.04 |
| 215449_at | BZRPL1 | 1.34 | 0.02 |
| 229617_x_at | AP2A1 | 1.30 | 0.03 |
| 209663_s_at | ITGA7 | 1.44 | 0.01 |
| 205780_at | BIK | 1.37 | 0.04 |
| 209680_s_at | KIFC1 | 1.36 | 0.03 |
| 207765_s_at | KIAA1539 | 1.36 | 0.03 |
| 209193_at | PIM1 | 1.30 | 0.02 |
| 219458_s_at | NSUN3 | 1.54 | 0.02 |
| 219654_at | PTPLA | 1.45 | 0.04 |
| 205919_at | HBE1 | 1.68 | 0.04 |
| 221870_at | EHD2 | 1.69 | 0.02 |
| 222634_s_at | TBL1XR1 | 1.52 | 0.04 |
| 209452_s_at | VTI1B | 1.47 | 0.05 |
| 209046_s_at | GABARAPL2 | 1.55 | 0.02 |
| 223084_s_at | CCNDBP1 | 1.33 | 0.04 |
| 202386_s_at | KIAA0430 | 1.34 | 0.02 |
| 201285_at | MKRN1 | 1.34 | 0.03 |
| 215499_at | LOC651423 | 1.44 | 0.02 |
| 227683_x_at |  | 1.51 | 0.04 |
| 217908_s_at | IQWD1 | 1.45 | 0.04 |
| 232349_x_at | IQWD1 | 1.58 | 0.02 |
| 59644_at | BMP2K | 1.54 | 0.02 |
| 219546_at | BMP2K | 1.55 | 0.02 |
| 243579_at | MSI2 | 1.54 | 0.05 |
| 235396_at | C22orf25 | 1.55 | 0.04 |
| 225319_s_at | FAM104A | 1.57 | 0.04 |
| 223124_s_at | C1orf128 | 1.58 | 0.02 |
| 208900_s_at | TOP1 | 1.49 | 0.04 |
| 210446_at | GATA1 | 1.58 | 0.05 |
| 231861_at | LRP10 | 1.69 | 0.02 |
| 221063_x_at | RNF123 | 1.51 | 0.02 |
| 1555590_a_at | GATA1 | 1.52 | 0.01 |
| 1552928_s_at | MAP3K7IP3 | 1.52 | 0.02 |
| 235582_at | E2F2 | 1.68 | 0.04 |
| 225061_at | DNAJA4 | 1.59 | 0.04 |
| 221246_x_at | TNS1 | 1.64 | 0.02 |
| 204147_s_at | TFDP1 | 1.61 | 0.05 |
| 223382_s_at | ZNRF1 | 1.49 | 0.04 |
| 202456_s_at | ZYG11BL | 1.48 | 0.03 |
| 209391_at | DPM2 | 1.60 | 0.02 |
| 201695_s_at | NP | 1.57 | 0.03 |
| 220320_at | DOK3 | 1.38 | 0.04 |
| 229373_at |  | 1.51 | 0.03 |
| 210589_s_at | GBA | 1.54 | 0.03 |
| 209093_s_at | GBA | 1.61 | 0.02 |
| 208052_x_at | CEACAM3 | 1.51 | 0.02 |
| 210102_at | LOH11CR2A | 1.47 | 0.03 |
| 206130_s_at | ASGR2 | 1.76 | 0.03 |
| 228412_at | LOC643072 | 1.80 | 0.04 |
| 211413_s_at | PADI4 | 1.80 | 0.03 |
| 235816_s_at | Rgr | 1.85 | 0.05 |
| 210610_at | CEACAM1 | 1.77 | 0.03 |
| 236979_at | C1orf178 | 1.93 | 0.02 |
| 229538_s_at | IQGAP3 | 2.19 | 0.02 |
| 243221_at | FAM20A | 1.76 | 0.02 |
| 233375_at | EFCAB2 | 2.01 | 0.04 |
| 223062_s_at | PSAT1 | 1.74 | 0.03 |
|  |  |  |  |
| Cluster IV |  |  |  |
|  |  |  |  |
| 221627_at | TRIM10 | 6.86 | 0.02 |
| 204187_at | GMPR | 6.03 | 0.01 |
| 231078_at |  | 6.95 | 0.01 |
| 222529_at | SLC25A37 | 5.65 | 0.01 |
| 222528_s_at | SLC25A37 | 7.06 | 0.01 |
| 231274_s_at |  | 5.21 | 0.00 |
| 226928_x_at | SLC25A37 | 6.09 | 0.00 |
| 228527_s_at | SLC25A37 | 7.95 | 0.00 |
| 221920_s_at | SLC25A37 | 6.54 | 0.00 |
| 205900_at | KRT1 | 6.62 | 0.03 |
| 204466_s_at | SNCA | 6.88 | 0.02 |
| 207827_x_at | SNCA | 6.12 | 0.01 |
| 211546_x_at | SNCA | 6.46 | 0.01 |
| 241881_at | OR2W3 | 7.01 | 0.02 |
| 224693_at | C20orf108 | 5.73 | 0.02 |
| 221748_s_at | TNS1 | 6.88 | 0.01 |
| 221747_at | TNS1 | 7.34 | 0.01 |
| 205837_s_at | GYPA | 5.55 | 0.02 |
| 1552583_s_at | ABCC13 | 6.15 | 0.02 |
| 223432_at | OSBP2 | 4.87 | 0.02 |
| 235683_at | SESN3 | 5.87 | 0.01 |
| 237299_at |  | 5.87 | 0.01 |
| 205389_s_at | ANK1 | 5.29 | 0.01 |
| 218644_at | PLEK2 | 6.03 | 0.02 |
| 210504_at | KLF1 | 7.69 | 0.01 |
| 228214_at |  | 7.78 | 0.01 |
| 233371_at | ABCC13 | 7.76 | 0.01 |
| 233267_at | SELENBP1 | 7.65 | 0.01 |
| 211820_x_at | GYPA | 8.54 | 0.01 |
| 207459_x_at | GYPB | 9.31 | 0.01 |
| 216398_at | GYPB | 7.83 | 0.01 |
| 216833_x_at | GYPB | 9.27 | 0.01 |
| 240336_at | HBM | 8.03 | 0.01 |
| 210088_x_at | MYL4 | 6.58 | 0.02 |
| 216054_x_at | MYL4 | 7.38 | 0.02 |
| 210395_x_at | MYL4 | 8.22 | 0.02 |
| 204848_x_at | HBG1 | 10.18 | 0.02 |
| 204419_x_at | HBG1 | 10.25 | 0.02 |
| 213515_x_at | HBG1 | 7.65 | 0.02 |
| 203911_at | RAP1GAP | 3.69 | 0.04 |
| 209735_at | ABCG2 | 3.47 | 0.03 |
| 205838_at | GYPA | 3.38 | 0.03 |
| 226599_at | KIAA1727 | 3.80 | 0.02 |
| 228251_at |  | 2.72 | 0.04 |
| 220757_s_at | UBXD1 | 2.62 | 0.04 |
| 222007_s_at | FKBP8 | 2.93 | 0.03 |
| 210430_x_at | RHD | 3.05 | 0.02 |
| 231933_at |  | 2.73 | 0.04 |
| 215037_s_at | BCL2L1 | 2.57 | 0.03 |
| 215812_s_at | SLC6A8 | 2.83 | 0.03 |
| 208353_x_at | ANK1 | 2.71 | 0.02 |
| 206303_s_at | NUDT4 | 2.83 | 0.02 |
| 201912_s_at | GSPT1 | 2.48 | 0.02 |
| 221479_s_at | BNIP3L | 2.82 | 0.02 |
| 207801_s_at | RNF10 | 3.10 | 0.01 |
| 227309_at | YOD1 | 2.52 | 0.02 |
| 204132_s_at | FOXO3A | 2.93 | 0.03 |
| 210655_s_at | FOXO3A | 2.91 | 0.02 |
| 227959_at |  | 2.96 | 0.04 |
| 218418_s_at | ANKRD25 | 3.21 | 0.04 |
| 212445_s_at | NEDD4L | 3.42 | 0.01 |
| 217889_s_at | CYBRD1 | 3.19 | 0.03 |
| 204720_s_at | DNAJC6 | 4.10 | 0.02 |
| 220173_at | C14orf45 | 3.58 | 0.02 |
| 213096_at | TMCC2 | 3.58 | 0.02 |
| 219814_at | MBNL3 | 2.88 | 0.04 |
| 227497_at |  | 3.56 | 0.02 |
| 1556283_s_at | FGFR1OP2 | 3.40 | 0.02 |
| 206302_s_at | NUDT4 | 3.73 | 0.02 |
| 209890_at | TSPAN5 | 4.14 | 0.02 |
| 225388_at | TSPAN5 | 3.88 | 0.02 |
| 212312_at | BCL2L1 | 3.93 | 0.02 |
| 225387_at | TSPAN5 | 3.26 | 0.02 |
| 221932_s_at | GLRX5 | 3.04 | 0.02 |
| 224789_at | WDR40A | 3.24 | 0.01 |
| 223266_at | ALS2CR2 | 3.03 | 0.01 |
| 203662_s_at | TMOD1 | 3.55 | 0.01 |
| 223669_at | HEMGN | 3.14 | 0.02 |
| 217274_x_at | MYL4 | 3.03 | 0.02 |
| 208255_s_at | FKBP8 | 2.89 | 0.02 |
| 40850_at | FKBP8 | 2.83 | 0.02 |
| 225800_at | tcag7.981 | 3.10 | 0.02 |
| 239205_s_at | CR1 | 2.94 | 0.01 |
| 228361_at | E2F2 | 2.94 | 0.01 |
| 218978_s_at | SLC25A37 | 3.14 | 0.01 |
| 205896_at | SLC22A4 | 3.26 | 0.01 |
| 242335_at | SLC25A37 | 3.79 | 0.00 |
| 218136_s_at | SLC25A37 | 4.14 | 0.00 |
| 223670_s_at | HEMGN | 4.23 | 0.04 |
| 218847_at | IGF2BP2 | 3.58 | 0.03 |
| 204505_s_at | EPB49 | 4.10 | 0.02 |
| 224690_at | C20orf108 | 4.23 | 0.02 |
| 215047_at | TRIM58 | 4.13 | 0.03 |
| 226811_at | FAM46C | 3.12 | 0.04 |
| 228770_at | GPR146 | 3.89 | 0.02 |
| 228634_s_at | CSDA | 2.93 | 0.03 |
| 201161_s_at | CSDA | 3.14 | 0.02 |
| 202947_s_at | GYPC | 3.31 | 0.03 |
| 210854_x_at | SLC6A8 | 3.46 | 0.02 |
| 213843_x_at | SLC6A8 | 3.74 | 0.02 |
| 202219_at | SLC6A8 | 3.66 | 0.02 |
| 239206_at | CR1L | 4.00 | 0.02 |
| 214273_x_at | C16orf35 | 3.30 | 0.02 |
| 218864_at | TNS1 | 3.87 | 0.02 |
| 1552713_a_at | SLC4A1 | 4.16 | 0.02 |
| 215150_at | YOD1 | 3.91 | 0.01 |
| 231982_at | LOC284422 | 4.04 | 0.01 |
| 215242_at | PIGC | 4.52 | 0.03 |
| 220807_at | HBQ1 | 4.12 | 0.01 |
| 223649_s_at | CGI-69 | 4.69 | 0.01 |
| 203116_s_at | FECH | 4.62 | 0.02 |
| 203502_at | BPGM | 4.97 | 0.01 |
| 241015_at |  | 4.59 | 0.01 |
| 235684_s_at | SESN3 | 4.84 | 0.01 |
| 203661_s_at | TMOD1 | 4.31 | 0.02 |
| 203115_at | FECH | 4.24 | 0.01 |
| 205856_at | SLC14A1 | 6.37 | 0.01 |
| 229151_at | SLC14A1 | 4.74 | 0.01 |
| 209116_x_at | HBB | 3.38 | 0.03 |
| 217232_x_at | HBB | 3.60 | 0.02 |
| 211696_x_at | HBB | 3.32 | 0.02 |
| 214414_x_at | HBA2 | 3.81 | 0.02 |
| 217414_x_at | HBA1 | 5.48 | 0.01 |
| 209458_x_at | HBA1 | 5.62 | 0.01 |
| 211745_x_at | HBA1 | 5.51 | 0.01 |
| 204018_x_at | HBA1 | 5.76 | 0.01 |
| 211699_x_at | HBA1 | 5.77 | 0.01 |
| 206522_at | MGAM | 3.31 | 0.05 |
| 206515_at | CYP4F3 | 3.82 | 0.03 |
| 203936_s_at | MMP9 | 5.40 | 0.03 |
| 209498_at | CEACAM1 | 4.70 | 0.01 |
| 209396_s_at | CHI3L1 | 4.76 | 0.02 |
| 209395_at | CHI3L1 | 8.34 | 0.01 |
| 209369_at | ANXA3 | 9.81 | 0.01 |
| 206851_at | RNASE3 | 3.69 | 0.05 |
| 203949_at | MPO | 4.02 | 0.02 |
| 210244_at | CAMP | 4.61 | 0.02 |
| 211657_at | CEACAM6 | 5.27 | 0.03 |
| 227140_at |  | 4.91 | 0.02 |
| 203021_at | SLPI | 4.96 | 0.01 |
| 205557_at | BPI | 5.66 | 0.02 |
| 212531_at | LCN2 | 7.19 | 0.01 |
| 208470_s_at | HP | 4.69 | 0.01 |
| 206697_s_at | HP | 5.26 | 0.01 |
| 207329_at | MMP8 | 8.16 | 0.02 |
| 231688_at |  | 9.37 | 0.01 |
| 206177_s_at | ARG1 | 7.28 | 0.02 |
| 207802_at | CRISP3 | 6.51 | 0.02 |
| 212768_s_at | OLFM4 | 12.12 | 0.01 |
| 205033_s_at | DEFA1 | 4.42 | 0.02 |
| 207269_at | DEFA4 | 7.68 | 0.02 |
| 206676_at | CEACAM8 | 7.34 | 0.02 |
| 202018_s_at | LTF | 10.39 | 0.01 |
| 211821_x_at | GYPA | 17.88 | 0.01 |
| 214407_x_at | GYPB | 20.51 | 0.01 |
| 219672_at | ERAF | 16.46 | 0.01 |
| 205592_at | IL8 | 17.69 | 0.01 |
| 214433_s_at | SELENBP1 | 12.22 | 0.01 |
| 210746_s_at | EPB42 | 14.70 | 0.01 |
| 205950_s_at | CA1 | 19.57 | 0.01 |
| 211560_s_at | ALAS2 | 23.89 | 0.01 |
| 206834_at | HBD | 22.66 | 0.00 |
