# Supplementary material for: Immature cell populations and an erythropoiesis gene-expression signature in systemic juvenile idiopathic arthritis: implications for pathogenesis
Source: Arthritis Res Ther. 2010 Jun 24;12(3):R123. doi: 10.1186/ar3061 (PMC2911917; doi:10.1186/ar3061)
Supplement: Additional file 4 — Receiver-operating characteristics (ROC) curve analysis by using the erythropoiesis index in a published cohort of patients with sJIA and other inflammatory conditions. The file contains figures showing (a) receiver-operating characteristics (ROC) curve analysis, comparing active sJIA (with fever) with bacterial infection and (b) ROC curve analysis, active sJIA (with fever) versus inactive sJIA (no fever). Gene-expression data published by Allantaz et al. [21] were retrieved from the Gene Expression Omnibus (GEO) database. Forty-nine probe sets were identical between the Affymetrix U133A (used by Allantaz et al.) and the U133 Plus 2.0 arrays (used by our group) and part of the erythropoiesis signature. The geometric mean of the linear expression values of these 49 probe sets was calculated for the individual samples, and the mean for the corresponding groups was calculated. AUC, area under the curve. [file ar3061-S4.DOC]

Additional file 4: Receiver-operating characteristics (ROC) curve analysis using the erythropoiesis index in a published cohort of patients with sJIA and other inflammatory conditions.


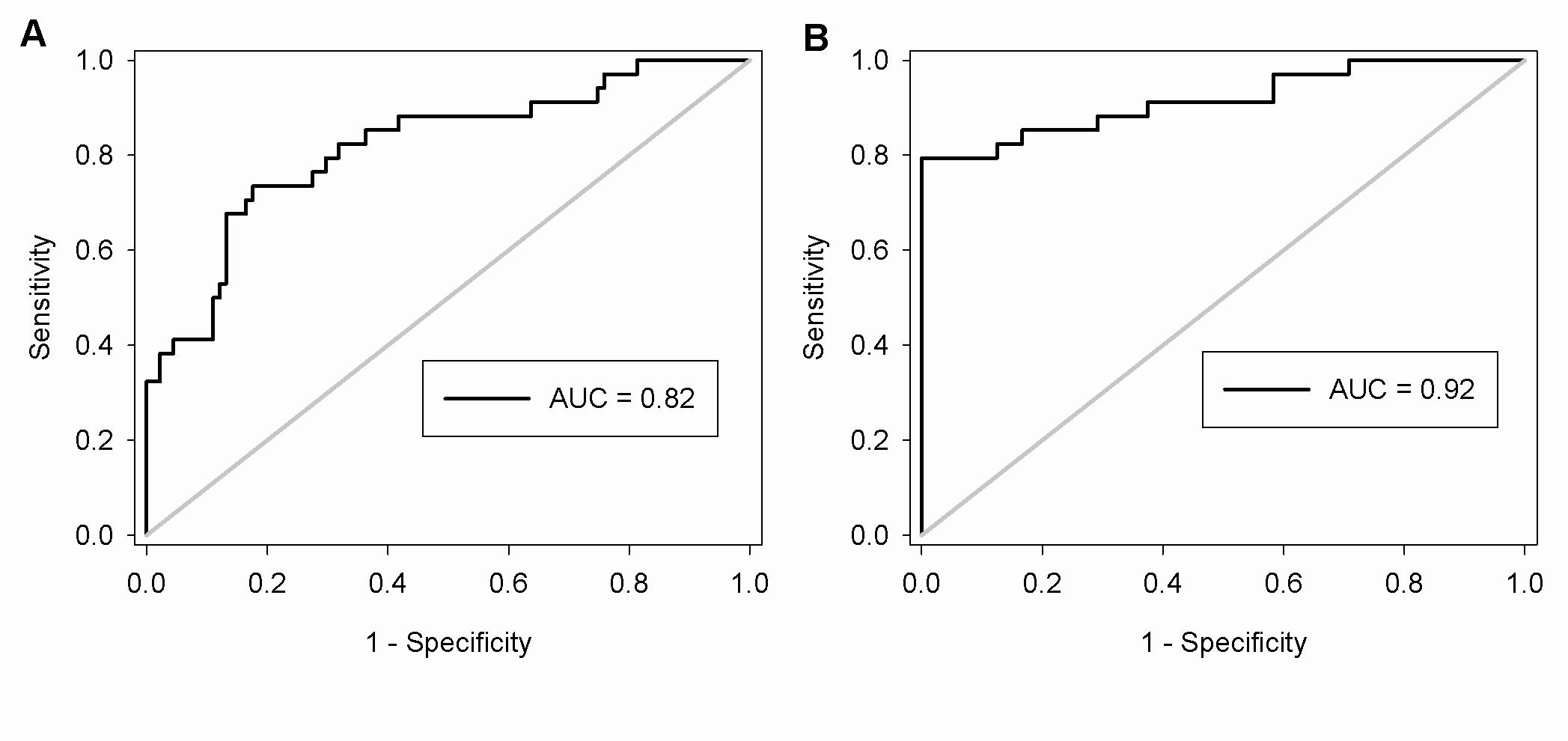


Gene expression data published by Allantaz et al. (21) were retrieved from the Gene Expression Omnibus (GEO) database. 49 probe sets were identical between the Affymetrix U133A (used by Allantaz et al.) and the U133 Plus 2.0 arrays (used by our group) and part of the erythropoiesis signature. The geometric mean of the linear expression values of these 49 probe sets was calculated for the individual samples and the mean for the corresponding groups was calculated. (A) Receiver-operating characteristics (ROC) curve analysis, active sJIA (with fever) versus bacterial infection, (B) ROC curve analysis, active sJIA (with fever) versus inactive sJIA (no fever). AUC, *are under the curve*
